# Proteomic Investigation of Glyceraldehyde-Derived Intracellular AGEs and Their Potential Influence on Pancreatic Ductal Cells

**DOI:** 10.3390/cells10051005

**Published:** 2021-04-24

**Authors:** Lakmini Senavirathna, Cheng Ma, Ru Chen, Sheng Pan

**Affiliations:** 1 The Brown Foundation Institute of Molecular Medicine, University of Texas Health Science Center at Houston, Houston, TX 77030, USA; herath.lakmini.senavirathna@uth.tmc.edu (L.S.); cheng.ma@uth.tmc.edu (C.M.); 2 Division of Gastroenterology and Hepatology, Department of Medicine, Baylor College of Medicine, Houston, TX 77030, USA; ru.chen@bcm.edu; 3 Department of Integrative Biology and Pharmacology, University of Texas Health Science Center at Houston, Houston, TX 77030, USA

**Keywords:** glycation, advanced glycation end products (AGEs), pancreatic cancer, glyceraldehyde, proteomics, mass spectrometry

## Abstract

Glyceraldehyde-derived advanced glycation end products (AGEs) play an important role in the pathogenesis of many diseases including cancer. Accumulation of intracellular AGEs could stimulate cancer induction and facilitate cancer progression. We evaluated the toxic effect of glyceraldehyde-derived intracellular AGEs on normal and malignant pancreatic ductal cells by assessing the cell viability, toxicity, and oxidative stress, followed by proteomic analysis. Our functional studies showed that pancreatic cancer cells (PANC-1 and MIA PaCa-2) were more resistant to glyceraldehyde treatment compared to normal pancreatic ductal epithelial cells (HPDE), while cytotoxicity effects were observed in all cell types. Furthermore, using ^13^C isotopic labeled glyceraldehyde, the proteomic data revealed a dose-dependent increment of the number of glycation adducts in both these cell types. HPDE cells showed a higher number of intracellular AGEs compared to cancer cells. At a molecular level, the glycations in the lysine residues of proteins showed a concurrent increase with the concentration of the glyceraldehyde treatment, while the arginine glycations appeared to be less affected by the glyceraldehyde doses. Further pathway analysis of these glycated proteins suggested that the glycated proteins participate in important biological processes that are major hallmarks of cancer initiation and progression, including metabolic processes, immune response, oxidative stress, apoptosis, and S100 protein binding.

## 1. Introduction

Pancreatic cancer is the third leading cause of cancer deaths in the USA [1]. Pancreatic ductal adenocarcinoma (PDAC) is the most common type of pancreatic cancer, as its occurrence is more than 90%. There are several risk factors associated with development of pancreatic cancer, such as age, genetics, cigarette smoke, obesity, dietary factors and diabetes mellitus (DM) [2,3,4,5]. Due to the poor prognosis of this disease, the 5-year survival rate is as low as below 10% [1,6]. Frequently, most of the PDACs are metastasized to other tissues or organs at the time of diagnosis. Therefore, its prevention and early diagnosis become important. Currently, the only clinical blood biomarker approved by the United States Food and Drug Administration for pancreatic cancer is cancer antigen 19-9 (CA 19-9), which is mostly used for monitoring the disease course during treatment [2].

There has been an increasing attention on advanced glycation end products (AGEs) for their implications in promoting cell dysfunction and disease onset [7,8,9], including PDAC [10]. AGEs are formed by non-enzymatic reactions between reducing sugar and proteins. For instance, reducing sugars such as glucose, fructose, galactose, mannose, and glyceraldehyde in the body can non-enzymatically react with amine groups of proteins, of which the mechanism is known as glycation. Glycation initially forms reversible Schiff base and Amadori products, which can further rearrange and form irreversible adducts known as AGEs [11]. These AGEs accumulate in our body either exogenously or endogenously [12,13]. The exogenous AGEs mainly depend on what we consume as foods. Foods that are rich in sugars and proteins, then processed at high temperatures, tend to form AGEs [11]. Endogenous AGEs are formed during breakdown of different reducing sugars within the body, or due to diseases, such as diabetes or hyperglycemia. Endogenous AGEs can be formed either extracellularly or intracellularly. Extracellular AGEs are primarily associated with glycation of blood proteins. This event is predominantly seen in diabetes patients which have a significant impairment of glucose tolerance and an increased accumulation of glucose in the blood [7]. On the other hand, due to the consumption of AGEs-rich meals, they can be absorbed into the blood stream during digestion [14]. Intracellular AGEs are formed during the glycation reaction between sugars and intracellular proteins [15]. Accumulation of AGEs in different tissues and organs in our body over time contributes to impaired cellular signaling, and thus, the development of different diseases, including cancer, cardiovascular diseases, and diabetic complications [12]. AGEs can bind to their receptors, known as RAGEs (Receptor for Advance Glycated End Products), and activate different cellular signaling to promote cancer hallmarks, such as inflammation, oxidative stress, proliferation, and invasion [9,16,17]. It has been shown in an in vitro study of prostate cancer that AGEs induced cell proliferation and invasion [18]. Furthermore, AGE treatment has shown to promote oxidative imbalances and inflammation in colorectal cancer cells [19]. *N*^ε^ -carboxymethyl lysisne (CML), one of the AGEs, was shown to increase the malignancy with metastasis in a pancreatic mice model [20].

The rate of the glycation differs with the types of sugars involved. Comparatively, glyceraldehyde is faster in glycation reactions than glucose [21,22]. Additionally, glyceraldehyde-derived AGEs are considered as toxic AGEs or TAGEs [23,24], and have been shown to promote pancreatic malignancies [10].

Based on current proteomic analyses, three types of glyceraldehyde-derived AGEs structures have been identified, namely, (i) glyceraldehyde-derived pyrinidium compound (GLAP), (ii) methylglyoxal-derived AGE or N^δ^-(5-hydro-5-methyl-4-imidazolon-2-yl)ornithine (MG-H1), and (iii) argpyrimidine (ArgP). GLAP occurs in lysine residues, while MG-H1 and ArgP occur in arginine residues of proteins [25,26,27].

Even though the cytotoxicity from glyceraldehyde-derived AGEs has been explored [10,24,28,29], there are limited information available on the identification of glyceraldehyde-derived intracellular AGEs by proteome-wide analysis [25]. In this study, we sought to understand the cytotoxic effect from glyceraldehyde-derived intracellular AGEs using different cellular assays, identify glyceraldehyde-derived protein AGE adducts by high resolution mass-spectrometry, and furthermore, discover potentially impaired functions of glycated proteins in normal and malignant pancreatic ductal epithelial cells through bioinformatics analysis.

## 2. Material and Method

### 2.1. Cell Culture and Glyceraldehyde Treatment

Human pancreatic ductal adenocarcinoma (PANC-1 and MIA PaCa-2, ATCC, Manassas, VA, USA) and immortalized normal human pancreatic ductal epithelial cells (HPDE-c7, gift from Dr. Xiaodong Cheng’s lab, UT Health Science Center at Houston, TX, USA) were seeded at 6000 cells/well in 96 well plates. PANC-1 and MIA PaCa-2 cells were maintained in DMEM (ATCC, Manassas, VA, USA) supplemented with 10% fetal bovine serum (FBS) and 1% Penicillin-Streptomycin. HPDE-c7 cells were maintained in Keratinocyte serum free medium supplemented with bovine pituitary extract and epidermal growth factor (Invitrogen, Carlsbad, CA, USA). The following day, the medium was removed and the fresh medium was added with the treatments as follows. Cells were treated with glyceraldehyde (Santa Cruz Biotechnology, Dallas, TX, USA) at final concentrations of 0, 1, 2, and 4 mM for 48 h at 37 °C. During the treatment, to achieve physiological conditions and prevent the effect from glycation of bovine serum, PANC-1 and MIAPaCa-2 cells’ culture media were switched to DMEM containing 10% human serum (Heat Inactivated human serum, Sigma, St. Louis, MO, USA) and 1% Penicillin-Streptomycin.

### 2.2. MTT Assay

After 48 h of the treatment, an MTT assay (ATCC, Manassas, VA, USA) was performed according to the manufacturer’s protocol. Absorbance values of glyceraldehyde-treated samples were normalized to the absorbance values of untreated control samples (0 mM).

### 2.3. LDH Activity

The cyQUANT^TM^ LDH assay (Invitrogen, Carlsbad, CA, USA) was performed according to the manufacturer’s protocol. The LDH release was calculated as a ratio of florescent values of treated samples over the fluorescent values of maximum LDH activity. The fluorescent values of glyceraldehyde treated samples were normalized to the fluorescent values of untreated controls (0 mM).

### 2.4. ROS Production

After 48 h of the treatment, the culture medium was removed and cells were washed with PBS three times. Then, 10 µM of 2′,7′-dichlorofluorescin diacetate (DCFH-DA) (Sigma, St. Louis, MO, USA) in DMEM (without FBS) and HBSS were added to PANC-1, MIA PaCa-2, and HPDE cells, respectively. Cells were incubated at 37 °C for 20 min followed by washing. Fluorescence of the 2′, 7′-dichlorofluorescin (DCF) was measured at the wavelengths of Ex/Em: 492/515 nm.

### 2.5. Western Blot

After 48 h of the treatment, the cells were washed with PBS two times and the cell lysate was collected with M-PER mammalian protein lysis buffer (Thermo Fisher Scientific, Waltham, MA, USA) containing 1% protease inhibitor (Sigma, St. Louis, MO, USA). Samples were sonicated followed by the centrifugation to remove cell debris. The protein concentration was measured using Bio-Rad protein assay dye reagent concentrate (Bio-Rad, Hercules, CA, USA). An equal number of proteins (10 µg for RAGE, GRP78, and Caspase-3, and 2 µg for β-actin) were loaded and separated in 10% or 12–20% gradient SDS-PAGE gels. Proteins were transferred to nitrocellulose membranes. The membranes were blocked with 5% non-fat milk. Then, they were probed with anti-RAGE mouse monoclonal antibody (1:300 dilution) (cat: sc-365154, Santa Cruz Biotechnology, Dallas, TX, USA), anti-GRP78 mouse monoclonal antibody (1:250) (Cat: sc-166490, Santa Cruz Biotechnology, Dallas, TX, USA), anti-Caspase-3 mouse monoclonal antibody (1:200) (Cat: sc-56053, Santa Cruz Biotechnology, Dallas, TX, USA), and anti-β-actin mouse monoclonal primary antibody (1:2000 dilution) (Cat. 3700T, Cell Signaling, Danvers, MA, USA) overnight at 4 °C with gentle shaking. After washing the membranes three times with TBS-T, the secondary antibody (peroxidase conjugated AffiniPure goat anti-mouse IgG, Jackson Immunoresearch, West Grove, PA, USA) was added (1:2000 dilution) and incubated for 1 h at room temperature with gentle shaking. The signal was generated by adding the Super Signal West Pico plus Chemiluminance substrate (ThermoFisher Scientific, Waltham, MA, USA) and visualized using KQ imager (Bio-Rad, Hercules, CA, USA). The western blot images were quantified using Image J software [30]. RAGE and GPR78 expressions were normalized to a reference gene (β-actin) and to the control (glyceraldehyde 0 mM).

### 2.6. Mass Spectrometric Analysis

The cells were treated with ^13^C_3_ stable isotopic labeled glyceraldehyde for 48 h as mentioned above in the method. Cell lysate was collected and homogenized and the cell debris was pelleted by centrifuging the lysate at 13,000 *g* for 15 min. The supernatant was collected and reduced with 10 mM DL-Dithiothreitol (DTT) at 50 °C for 1 h. Then, samples were alkylated with 25 mM iodoacetamide at room temperature for 30 min in the dark. Tricholoracetic acid (TCA) precipitation was performed by adding one fourth volume of 100% (*w*/*v*) tricholoracetic acid. Samples were incubated on ice for 10 min and centrifuged at 14,000 *g* for 5 min at 4 °C. The precipitate was washed with ice-cold acetone twice and air dried. The precipitate was suspended in 50 mM ammonium bicarbonate. Proteins were digested with Trypsin (1:30) or Glu-C in a two-step process. In the first step, half the amount of the enzyme was added and the samples were incubated at 37 °C for 2 h with vortexing at every 30 min. Then, the other half of the enzyme was added and the samples were incubated at 37 °C for overnight. After the enzymatic digestion, the reaction was stopped by adding 1 volume of 1% formic acid. Samples were dried at room temperature in a speed vacuum concentrator, and further purified using C18 columns (BioPureSPN™ MACRO TARGA^®^ C18, Nest Group, Southborough, MA, USA). The eluent was dried in a speed vacuum concentrator and suspended in 0.1% formic acid. One microgram of digested samples was analyzed with a Q Exactive™ HF-X Orbitrap ™ mass spectrometer (Thermofisher, Waltham, MA, USA) interfaced with an UltiMate 3000 HPLC (Thermofisher, Waltham, MA, USA). The samples were first loaded into a 5-mm trap column packed with 5 µM/100 Å C18 material (Thermofisher, Waltham, MA, USA) using 98% buffer A (0.1% formic acid in water) and 2% buffer B (0.1% formic acid in acetonitrile) at a flow rate of 5 µL/min. The samples were separated in a 25 cm analytical column packed with 5 µM/18 Å C18 material using a 90 min linear gradient from 2% to 35% buffer B versus buffer A at a flow rate of 0.35 µL/min. Mass spectrometric analysis was performed using data dependent acquisition (DDA) mode with a *m/z* range of 400–1600, consisting of a full MS scan followed by up to 25 MS/MS spectra acquisitions in the Orbitrap using higher energy collisional dissociation (HCD).

### 2.7. Data Analysis

The generated raw files were converted to mzML format and searched against the Uniprot human database using Comet algorithm (2019.01 rev. 0 version) embedded in Trans Proteomic Pipeline (TPP, version v5.1.0). The database search was restricted with the following modification parameters, including: cysteine carboxamidomethylation (fixed), methionine oxidation (variable), lysine AGE modification-GLAP (variable), arginine AGE modification-ArgP (variable), and arginine AGE modification-MG-H1 (variable). Peptide identification was validated using PeptideProphet with 1% false discovery rate (FDR).

### 2.8. Functional and Statistical Analysis

Functional annotations (gene ontology (GO), KEGG pathway and Reactome pathway) of AGE-modified proteins were performed using STRING version 11.0 [31]. Statistical analysis was performed by ANOVA with Fisher’s LSD multiple comparison post-hoc test using GraphPad Prism.

## 3. Results

### 3.1. Effect of Glyceraldehyde Treatment on Cell Viability

**Figure 1:**
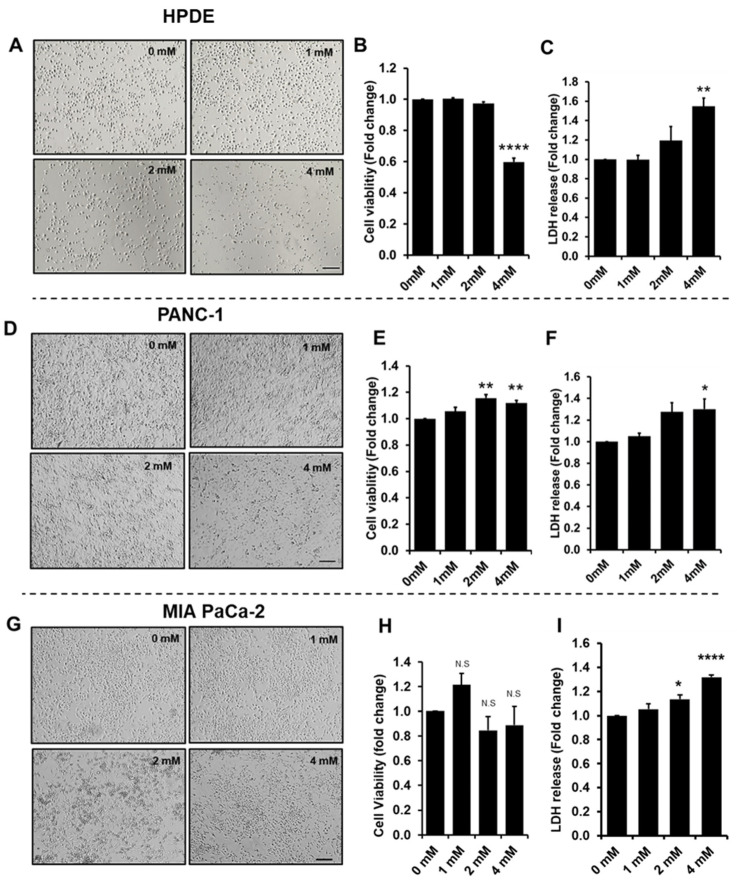
HPDE, PANC-1, and MIA PaCa-2 cells treated with glyceraldehyde for 48 h. (**A**,**D**,**G**) Bright field images of cells treated for 48 h with glyceraldehyde. Scale bar: 200 µm. (**B**,**E**,**H**) Cell viability determined by MTT assay. (**C**,**F**,**I**) Cell damage determined by LDH assay. Absorbance or fluorescence values were normalized to control (0 µM). Data represent the mean (*n* = 3) ± SE. N.S, not significant, **** *p* < 0.0001, ** *p* < 0.01, * *p* < 0.05 vs. control (0 mM).

PANC-1, MIA PaCa-2, and HPDE cells were treated with 1, 2, and 4 mM of glyceraldehyde for 48 h, and cell viability and cytotoxicity were determined by MTT assay and LDH assay, respectively. The treatment with the highest concentration (4 mM) of glyceraldehyde showed adverse effect on cell viability of HPDE cells. The bright field images of the HPDE cells indicated that the cell number was greatly reduced after the treatment (Figure 1A), and the cell viability of HPDE was lost by about 40% compared to the control with no treatment (Figure 1B). On the other hand, PANC-1 and MIA PaCa-2 cells responded differently to the glyceraldehyde treatment for the given doses. The PANC-1 cells showed a slightly increased in cell viability (15–20%) during 2 and 4 mM of glyceraldehyde treatment (Figure 1D,E) and the viability of MIA PaCa-2 cells were not significantly affected by glyceraldehyde treatment (Figure 1G,H). It appeared that HPDE cells were more vulnerable to lose the viability at higher glyceraldehyde concentrations. Furthermore, the cytotoxicity test indicated that all these cells had an increased LDH release during the 2 mM and 4 mM treatments (Figure 1C,F,I). The increased level of LDH release in HPDE at 4 mM treatment (increase of 64%) appeared to be much higher compared to the LDH release of PANC-1 and MIA PaCa-2 cells (increase of 20–30%) at the same treatment dose. These observations might suggest that the glyceraldehyde treatment potentially caused a cytotoxic effect on all these cells due to the formation of protein AGEs; however, for the given doses, the cytotoxic effect in PANC-1 and MIA PaCa-2 cells was not enough to affect their viability, and cells might switch their cellular mechanisms to increase the proliferation in PANC-1 cells or sustain the viability of MIA PaCa-2 cells [10].

### 3.2. Increased Oxidative Stress during Glyceraldehyde Treatment

**Figure 2:**
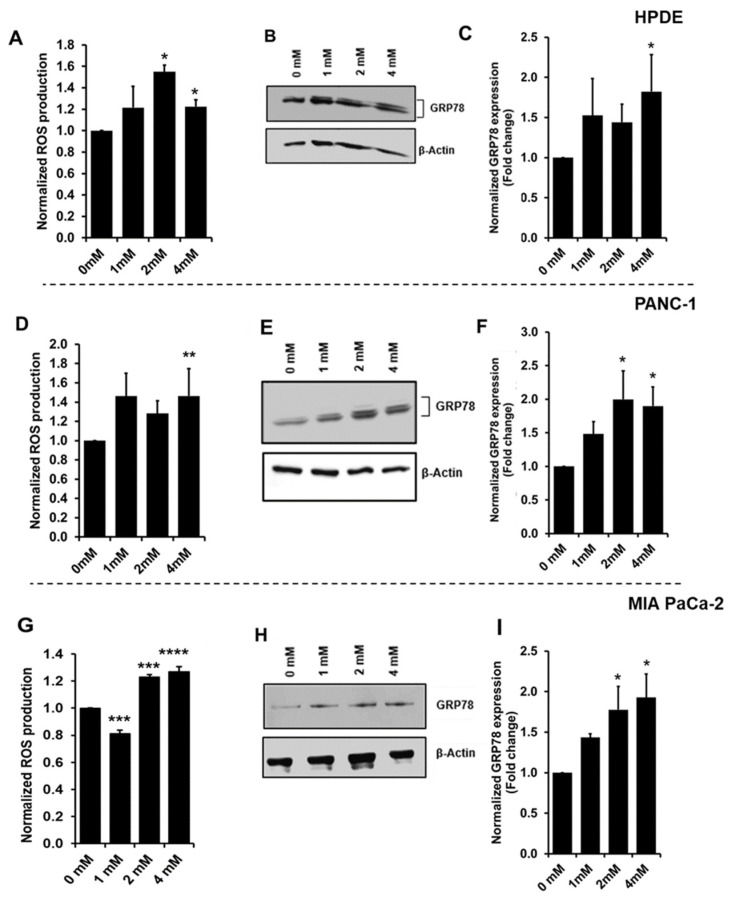
Oxidative Stress in HPDE, PANC-1, and MIA PaCa-2 cells treated with glyceraldehyde for 48 h. (**A**,**D**,**G**) ROS production determined as fluorescence of 2′, 7′-dichlorofluorescin (DCF) production. Data were normalized to cell viability. (**B**,**E**,**H**) Western blot for GRP78 expression in cells treated with glyceraldehyde. (**C**,**F**,**I**) Quantitative analysis of GRP78 expression in cells treated with glyceraldehyde. Data were normalized to control (0 µM). Data represent the mean (*n* = 3) ± SE. **** *p* < 0.0001, *** *p* < 0.001, ** *p* < 0.01, * *p* < 0.05 vs. control (0 mM).

The oxidative stress from the glyceraldehyde treatment on cells was evaluated by the reactive oxygen species (ROS) production and the protein expression of GRP78. The ROS production was determined by the fluorescence production of 2′,7′-dichlorofluorescin (DCF). After the glyceraldehyde treatment, 2′,7′-dichlorofluorescin diacetate (DCFH-DA) was added. DCFH-DA is a cell-permeable substance and the intracellular ROS converts DCFH-DA to fluorogenic DCF [32]. Elevated fluorescent values were observed with the glyceraldehyde treatment, which indicated the increased intracellular ROS production. Since HPDE cells showed a greater loss of cell viability (Figure 1B) and also the loss of the cell number (Figure 1A), the ROS production was normalized to the cell viability in each cell type. HPDE, PANC-1, and MIA PaCa-2 cells showed increased ROS production against the treatment of the higher glyceraldehyde concentrations (Figure 2A,D,G). The protein expression of GRP78 (also known as BiP or HSPA5), which is known to be induced during oxidative stress or ER stress [33], was also determined by western blot. As indicated in Figure 2B,C (HPDE), Figure 2E,F (PANC-1), and Figure 2H,I (MIA PaCa-2), all the glyceraldehyde treatments induced the GRP78 expression in the cells, suggesting that both normal and malignant cells were under a type of stress during the treatments.

### 3.3. Glyceraldehyde Treatment Induced the Apoptosis in HPDE Cells

**Figure 3:**
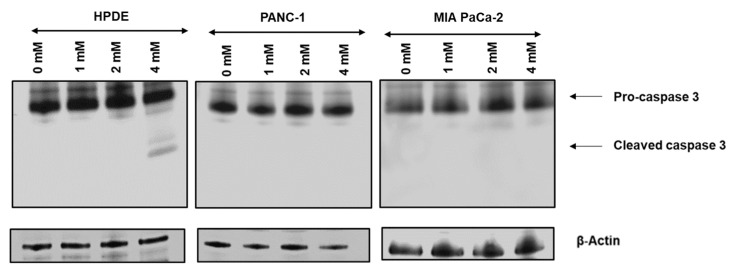
Western blot analysis of cleaved caspase-3 in HPDE, PANC-1, and MIA PaCa-2 cells treated with glyceraldehyde. Western blot was performed for three independent experiments for each cell type.

Bright filed cell images and MTT assay (Figure 1) showed that HPDE cells lose the cell number and viability in significant levels at 4 mM glyceraldehyde treatment, while the viability of PANC-1 and MIA PaCa-2 cells was not severely affected by the glyceraldehyde treatments. To test the cells undergoing apoptosis during the treatment, western blot was performed to detect the apoptosis marker cleaved caspase-3. Noticeably, caspase-3 was activated and cleaved only in HPDE treated with 4 mM glyceraldehyde (Figure 3), confirming that HPDE cells are more susceptible to glyceraldehyde treatment compared to PANC-1 and MIA PaCa-2 cells.

### 3.4. Glyceraldehyde Treatment Induced the RAGE Expression

**Figure 4:**
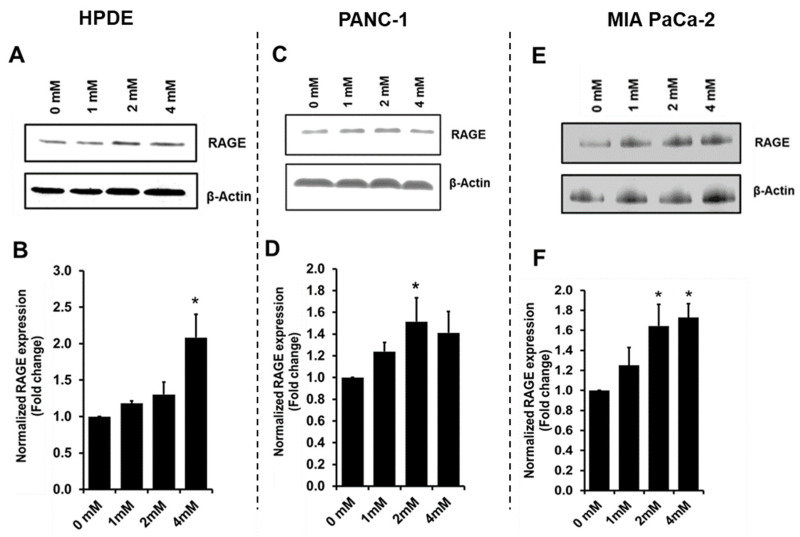
RAGE expression in HPDE, PANC-1, and MIA PaCa-2 cells treated with glyceraldehyde for 48 h. (**A**,**C**,**E**) Western blot for RAGE expression in cells treated with glyceraldehyde. (**B**,**D**,**F**) Quantitative analysis of RAGE expression in cells treated with glyceraldehyde. Data were normalized to control (0 µM). Data represent the mean (*n* = 3) ± SE. * *p* < 0.05, vs. control (0 mM).

RAGE is the receptor for AGE ligands, and AGE-RAGE binding is known to increase the RAGE expression, which can stimulate a cascade of cancer associated inflammatory pathways and protein network activities [13,34,35,36]. Using western blot, we observed that the RAGE expression was increased (Figure 4) with the glyceraldehyde treatments for HPDE, PANC-1, and MIA PaCa-2 cells. Using the control with no treatment as a background, at 4 mM glyceraldehyde treatment, HPDE cells showed a greater increase of RAGE expression (100%) compared to PANC-1 cells (40%) and MIA PaCa-2 (70%).

### 3.5. Identification of Intracellular AGEs Formed by Glyceraldehyde Treatment

**Figure 5:**
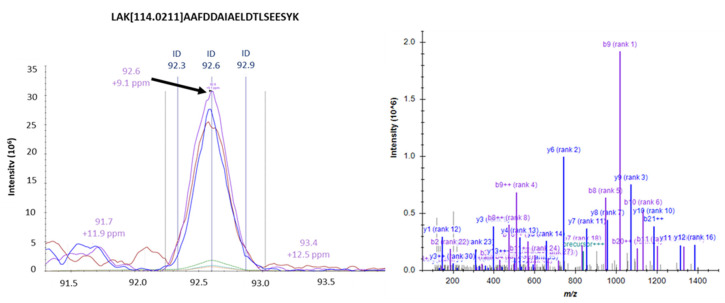
Elution profile and spectral identification of a K-glycated AGE peptide identified by high-resolution mass spectrometry.

**Figure 6:**
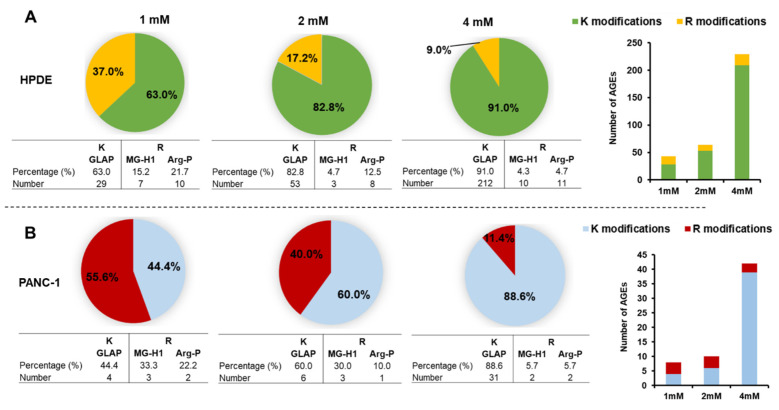
Mass spectrometric identification of ^13^C_3_-glyceraldehyde derived K/R AGE modifications in cellular proteins of HPDE (**A**) and PANC-1 (**B**) cells. GLAP: Glyceraldehyde derived pyridinium type advanced glycation end product, Arg-P: Argpyrimidine, MG-H1: *N^δ^*-(5-hydro-5-methyl-4-imidazolon-2yl) ornithine.

**Figure 7:**
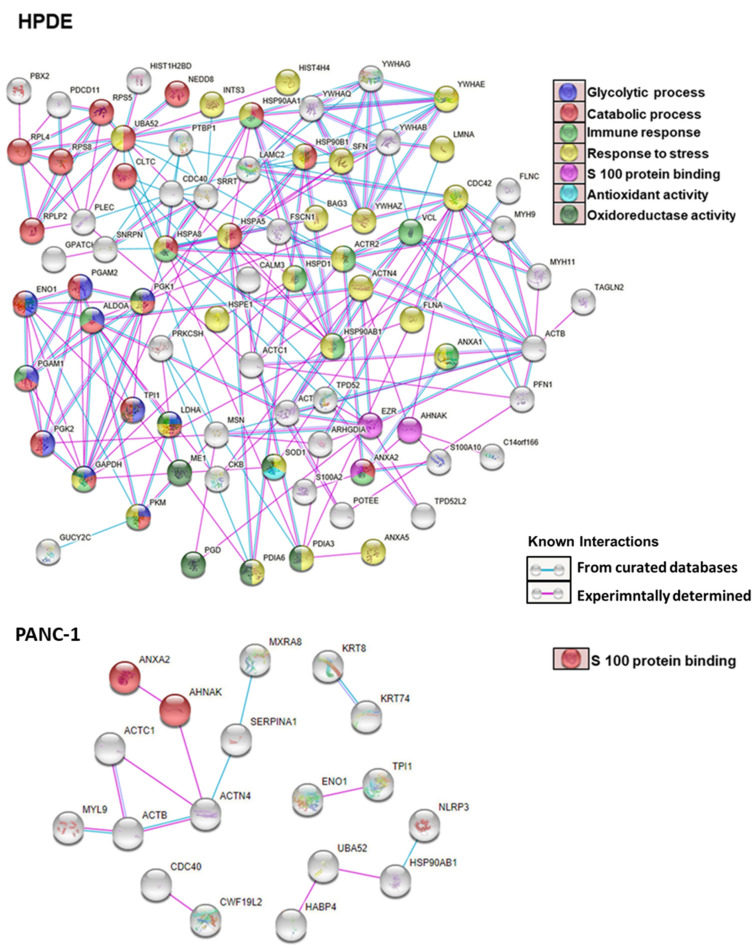
Protein–protein interactions (PPI) of proteins with glyceraldehyde-derived K/R modifications.

To identify the specific AGE forms, we used mass spectrometry to analyze the cell protein lysates of HPDE and PANC-1. As shown in Tables S1 and S2, we found various AGEs formed in the intracellular proteins upon treatment of glyceraldehyde. The glyceraldehyde-derived AGEs include lysine-conjugated GLAP, as well as arginine-conjugated MG-H1 and ArgP. Figure 5 exemplifies the mass spectrometric identification and quantification of a protein site-specific GLAP. A larger number of intracellular AGEs were identified in HPDE cells (*n* = 104) compared to PANC-1 cells (*n* = 28) (Figure S1). Among these glycated proteins, 11 proteins were common for AGEs modification between HPDE and PANC-1 (Table S3). Interestingly, we found that lysine glycations were increased concurrently with the glyceraldehyde concentrations in both HPDE and PANC-1 cells, while the number of arginine modifications remained less affected (Figure 6). Furthermore, some peptides were particularly vulnerable for glycation and subjected to multiple AGEs, possibly due to their specific sequences and local neighboring environment (Tables S4 and S5). The protein–protein interactions of the AGE-modified proteins are illustrated in Figure 7. In HPDE cells, which appeared to be more vulnerable for intracellular AGE formation, the glycated proteins were implicated in multiple protein networks relevant to cancer promotion, including glycolytic and catabolic processes, immune response, S100 protein binding, and antioxidant and oxidoreductase activities. On the other hand, while the influence of glyceraldehyde treatment was substantially less on PANC-1 cells, proteins involved in inflammatory pathways were glycated intracellularly.

### 3.6. Gene Ontology and Pathway Analysis

Molecular functions in GO analysis (Figures S3 and S4) showed that AGE-modified proteins (Tables S6 and S7) in both HPDE and PANC-1 cells are involved in S100 protein binding. Three glycated proteins belonging to this groups were Annexin A2, Neuroblast differentiation-associated protein AHNAK, and Ezrin. Annexin A2 and AHNAK proteins were glycated in both HPDE and PANC-1 cells, while Ezrin was only found to be glycated in HPDE cells. Besides S 100 protein binding, glycated proteins of HPDE cells are involved in additional networks that are cancer relevant, including immune response, glycolytic process, catabolic process, response to stress, antioxidant, and oxidoreductase activity (Table S8). Furthermore, pathway analyses showed that the glycated proteins were involved in glycolysis carbon metabolism, proteoglycans in cancer, carbon metabolism in cancer, immune response, transcriptional regulation by TP3, apoptosis, and DNA damage checkpoints (Table S8).

## 4. Discussion

Glyceraldehyde is an important intermediate product of sugar metabolism. During glycolysis, glucose is converted into glyceradhyde-3-phosphate. It can be further non-enzymatically converted to glyceraldehyde through de-phosphorylation. Additionally, fructose can also be metabolized to glyceraldehyde through fructolysis by fructokinase and aldolase [11,37]. It has been shown from different studies that glyceraldehyde-derived AGEs promote carcinogenesis [10,24,28].

In this study, we first investigated the cytotoxic effect from glyceraldehyde on normal and malignant pancreatic ductal epithelial cells in the context of intracellular AGE formation. The cell viability was negatively affected in normal (HPDE) cells, while malignant (PANC-1 and MIA PaCa-2) cells did not show adverse influence in proliferation with the glyceraldehyde treatment (Figure 1). Notably, a previous study has shown that under hyperglycemic conditions, a glyceraldehyde treatment adversely affected PANC-1 cells’ viability [10]. This difference in observation could be due to the different culturing conditions. In our study, PANC-1 cells were cultured with 10% human serum (by replacing bovine serum) without hyperglycemic conditions. Additionally, differences in metabolic pathways between normal and malignant cells could be one reason that HPDE cells were more vulnerable, while PANC-1 and MIA PaCa-2 cells were more favorable for the growth upon the given glyceraldehyde treatments. To further confirm this observation, PANC-1 and MIA PaCa-2 cells were treated in serum free condition for 48 h, and their viabilities were not adversely affected during the treatments (Figure S2). PANC-1 and MIA PaCa-2 cells are malignant cells with altered metabolism and they might proliferate by utilizing glyceraldehyde as glyceradehyde-3-phospahate. The LDH activity of all these cells were induced by the glyceraldehyde treatment and it was higher in HPDE cells than in PANC-1 and MIA PaCa-2 cells (Figure 1). When cells are under a type of stress, they may switch to anaerobic glycolysis for energy production and, as a result, the activity of LDH would be increased. PANC-1 and MIA PaCa-2 cells also showed a slightly increased LDH activity, and they might also utilize both aerobic and anaerobic glycolysis to achieve efficient energy consumption during the glyceraldehyde treatment. Furthermore, cancer cells favor hypoxic conditions for their growth, and the increased LDH activity could be due to the Warburg effect [38].

Oxidative stress is involved in many physiological functions including proliferation, apoptosis, tumorigenesis, and activation of different cellular signaling. The level of ROS production determines which biological processes are activated. It is known that low ROS levels promote the cell proliferation and growth, while higher ROS levels induce apoptosis [39,40]. A study conducted on PANC-1 cells using a high glucose treatment showed that cell proliferation and ROS production were increased while inhibition of ROS production in these cells reduced the colony formation [41]. In contrast, another study has shown that an increased ROS level induced the apoptosis in PANC-1 cells via ROS-induced ERK activation [42]. In our experiments, the glyceraldehyde treatment increased the ROS production in all of these three cells (Figure 2). The level of ROS in PANC-1 and MIA PaCa-2 cells was not high enough to reduce the cell viability (Figure 1E,H). However, it was apparent that the ROS production in HPDE cells during the treatment negatively affected the cell viability, as we indicated by using an MTT assay (Figure 1B). Even though we expected the highest ROS production to be occurred in HPDE at 4 mM glyceraldehyde treatment, the highest observed ROS production actually took place in 2 mM glyceraldehyde treatment. In our study, ROS production was normalized to cell viability, since the cell number was substantially reduced in HPDE cells at 4 mM glyceraldehyde treatment. As such, due to the loss of the cell number and viability, a greater proportion of intracellular ROS production was not being able to detect at higher glyceraldehyde concentration. To further evaluate the oxidative stress in these cells, we examined the protein expression of GRP78, which is known to be induced during oxidative stress or ER stress. Indeed, the GRP78 expression was induced by the glyceraldehyde treatment in all three types of cells (Figure 2). The role of GRP78 during ER stress is important, as it acts as an ER chaperon and participates in the degradation of misfolded proteins in ER lumen [33]. A study reported that silencing of GRP78 significantly reduced the tumor progression and cell viability in a xenograft (PANC-1) mice model and PANC-1 cells, respectively [43]. Additionally, it is known that moderately elevated ROS induce oncogenes and inhibit tumor suppressor genes [44,45,46]. Certain levels of reactive oxygen species can promote cell proliferation, particularly in cancer [40]. These findings suggest that ROS and GRP78 are implicated in tumor progression, which is consistent with our results of PANC-1 and MiaPac-2, in which increased ROS and GRP78 expression promoted the cell survival and growth during the glyceraldehyde treatment. We observed a significant loss of cell viability in HPDE cells at 4 mM glyceraldehyde treatment; activated caspase-3 was detected by western blot, confirming this observation (Figure 3). As expected, cleaved caspase-3 was detected in HPDE cells treated with 4 mM glyceraldehyde, but neither with other doses nor other cells.

We further examined the expression of RAGE as the potential consequence of intracellular AGE formation. RAGE was increased in all the three types of cells after glyceraldehyde treatment, especially at higher concentrations (Figure 4). Several studies have reported that RAGE expression could be induced by AGE formation in different cell types [20,36,47,48]. The accumulation of ligands and inflammatory mediators can stimulate RAGE expression [34,49]. In this study, we sought to assess the effect of intracellular AGE formation on RAGE expression, which was found sensitive in response to glyceraldehyde treatment, even at low concentrations. However, this effort could be compromised by a limitation, of which the glyceraldehyde treatment could also potentially form extracellular AGEs with either serum proteins or growth factors in the media. While it was difficult to de-convolute the effect of possible extracellular AGEs, the dose-dependent induction of RAGE expression might imply potential intracellular molecular events implicated in increased RAGE expression and RAGE ligand formation.

Several studies have shown that extracellular AGEs impacted on different cellular functions, such as cell proliferation [20,47,50], migration [36], increased ROS production, and TGFβ signaling [48]. Additionally, the lower AGEs concentration increased the cell proliferation while the higher doses inhibited it [50]. Therefore, in our study, the intracellular AGEs formation has a potential role for these observed effects. At higher doses of glyceraldehyde treatment, the relative AGEs accumulation was higher in HPDE compared to cancer cells, and at the same time, cell death was observed in HPDE. The possible mechanism could be that intracellular AGEs secreted extracellularly or released during cell death and binds with RAGEs of surrounding cells and activate different cellular signaling pathways. Besides AGEs itself, stimulated RAGE expression also contributes to cell proliferation. Overexpression of RAGE in pancreatic cells showed increased cell proliferation, and silencing reduced the RAGE-induced proliferation [51]. In addition, other cells also showed cell proliferation, corresponding with RAGE expression [52,53]. The role and mechanism of intracellular AGEs in the noted cellular effects and RAGE expression still remains unclear and is yet to be demonstrated.

The proteomic data in our study revealed a larger number of protein AGE adducts in HPDE cells compared to PANC-1 cells. This is consistent with our cellular experiments, in which we observed that HPDE cells were more vulnerable with glyceraldehyde treatment compared to PANC-1. Glyoxalase 1 is an enzyme which detoxifies the methylglyoxal (MG) precursor of AGEs [54,55,56]. This enzyme is highly expressed in tumors and cancerous tissues compared to healthy tissues [57,58]. Our results confirmed that PANC-1 cells have less AGEs accumulation compared to normal HPDE cells, possibly due to higher expression of glyoxalase 1. Additionally, an increased production of MG is responsible for RAGE signaling and oxidative stress [59], suggesting that HPDE cells were more vulnerable to intracellular AGE accumulation. The proteomic data further indicated that lysine glycations were concurrently increased with the glyceraldehyde concentrations, whereas the dose effect on arginine glycations was negligible (Figure 6). It has been reported that the occurrence of glyceraldehyde- and glyoxal-derived lysine glycations are higher than arginine glycations in serum albumin and mouse collagen [60,61]. Furthermore, lysine-based AGEs, such as CML, have shown to be involved in the biology of cancers by activating transcription factor NF-kB [62] and upregulating VEGF [63] to promote angiogenesis and induce DNA damage [64], leading to cancer progression. Our observation demonstrated the notion that lysine was more liable to a dose-dependent AGE modification, likely due to the nature of its chemical structure. The surrounding amino acids are in a hydrophobic and acidic environment, which is likely to be important in determining the glycation site [65]. This observation, if further validated, might have an important implication in developing novel strategies in reducing the formation and accumulation of toxic AGEs in cancer prevention.

Although protein glycation and AGE formation are often viewed as proteome-wide phenomena, certain proteins which are more vulnerable to glycation could be involved in cellular pathways related to tumorigenesis. The functional clustering analysis of the proteomic data suggested that the glycated proteins were involved in many protein networks and pathways relevant to pancreatic cancer, including the metabolic processes, immune response, oxidative stress, apoptosis, and S100 protein binding. Additionally, there is a higher probability that these glycation sites could be the same for other post-translational modifications, such as ubiquitination or acetylation; thus, glycation could impair the protein stability and gene expression [65]. Further investigations are required to demonstrate how these AGEs formation could influence the functions of glycated proteins and impact carcinogenesis.

## 5. Conclusions

In summary, the glyceraldehyde treatment on pancreatic ductal epithelial cells differentially affected the formation of intracellular AGEs in pancreatic ductal normal (HPDE) and cancer (PANC-1, MIA PaCa-2) cells, with HPDE cells being more vulnerable to the glyceraldehyde-derived AGE toxicity. Consistently, at a proteome level, there is a higher chance of glycation of proteins in normal cells than pancreatic cancer cells. Lysine residues are more susceptible to get glycated through glyceraldehyde than arginine. The glycated proteins were involved in many networks relevant to cancer promotion and progression, including metabolism, immune response, oxidative stress, apoptosis, and S100 protein binding. It is remarkable that we observed that lysine residues were significantly more vulnerable for glyceraldehyde-derived AGE formation compared to arginine residues, an important finding that may have potential implication for prevention and treatment of AGE-dependent diseases, including cancer. Future investigations are warranted to further validate the functional impairments attributed to AGE formation under various conditions and investigate their mechanistic roles in AGE related disease, especially cancer.

## Data Availability

The data presented in this study are available on a reasonable request from the corresponding author.

## Supplementary Materials

The following are available online at https://www.mdpi.com/article/10.3390/cells10051005/s1: Figure S1. Venn diagram showing the number of modified proteins through glyceraldehyde treatment, Figure S2. PANC-1 and MIA PaCa-2 cells treated with glyceraldehyde for 48 h in serum free media., Figure S3. Functional annotations of AGEs modified proteins in glyceraldehyde treated HPDE cells. (A) GO analysis-Biological Processes. (B) GO analysis-Molecular Functions. (C) GO analysis-Cellular components. (D) KEGG pathway analysis. (E) Reactome Pathway analysis, Figure S4. Functional annotations of AGEs modified proteins in glyceraldehyde treated PANC-1 cells. (A) GO analysis-Molecular functions. (B) GO analysis-Cellular components. (C) KEGG pathway analysis. (D) Reactome Pathway analysis. Table S1. Peptide list of HPDE with AGE modifications, Table S2. Peptides list of PANC-1 with AGE modifications, Table S3. The 11 shared proteins between PANC-1 and HPDE, Table S4. Peptides with multiple AGEs in HPDE treated with glyceraldehyde, Table S5. Peptides with multiple AGEs in PANC-1 treated with glyceraldehyde, Table S6. Glycated proteins in HPDE cells, Table S7. Glycated proteins in PANC-1 cells, Table S8. Glycated proteins in HPDE cells involved in different functions.
