# Supplementary material for: Proteomic Investigation of Glyceraldehyde-Derived Intracellular AGEs and Their Potential Influence on Pancreatic Ductal Cells
Source: Cells. 2021 Apr 24;10(5):1005. doi: 10.3390/cells10051005 (PMC8145644; doi:10.3390/cells10051005)
Supplement: Supplementary file 1 [file cells-10-01005-s001.zip › Supp_files_proofread/Sup tables _proof read.pdf]

Table S1. Peptides list of HPDE having AGEs modifications

AGEs	Peptide	Accession number	Protein	Protein description	Probability
GLAP	R.LAK[242.12]AAFDDAIAELDTLSEESYK.D	P62258	1433E	14-3-3 protein epsilon	0.9811
GLAP	R.AEGSDVANAVLDGADC[160.03]IMLSGETAK[242.12]GDYPLEAVR.M	P14618	KPYM	Pyruvate kinase PKM	0.9973
GLAP	K.AGYTDK[242.12]VVIGMDVAASEFFR.S	P06733	ENOA	Alpha-enolase	0.9937
GLAP	R.SVFLGK[242.12]IK[242.12]DAFDRNPELQNLLLDDFFK.S	P52209	6PGD	6-phosphogluconate dehydrogenase, decarboxylating	0.9918
GLAP	K.AC[160.03]SLAK[242.12]TAFDEAIAELDTLSEESYK.D	P63104	1433Z	14-3-3 protein zeta \ delta	0.9977
GLAP	K.ELASALK[242.12]SALSGHLETVILGLLK.T	P07355	ANXA2	Annexin A2	1
GLAP	K.YLMIEEYLTK[242.12]ELLALDSVDPEGR.A	O95817	BAG3	BAG family molecular chaperone regulator 3	0.9102
GLAP	R.GSPGPPENVK[242.12].V	Q9P232	CNTN3	Contactin-3	0.9996
GLAP	K.IISNASC[160.03]TTNC[160.03]LAPLAK[242.12]VIHDNFGIVEGLMTTVHAITATQK.T	P04406	G3P	Glyceraldehyde-3-phosphate dehydrogenase	1
GLAP	K.K[242.12]GK[242.12]QEEEKPGEEK.T	O60508	PRP17	Pre-mRNA-processing factor 17	0.9992
GLAP	K.DSYVGDEAQSK[242.12]R.G	P68032	ACTC	Actin, alpha cardiac muscle 1	1
GLAP	K.LDPK[242.12]IAVAAQNC[160.03]YK.V	P60174	TPIS	Triosephosphate isomerase	1
GLAP	R.VAPEEHPVLLTEAPLNPK[242.12]ANR.E	P60709	ACTB	Actin, cytoplasmic 1	0.9997
GLAP	K.K[242.12]QELEEIC[160.03]HDLEAR.V	P35579	MYH9	Myosin-9	1
GLAP	K.TLTGK[242.12]TITLEVEPSDTIENVK.A	P62987	RL40	Ubiquitin-60S ribosomal protein L40	0.9979
GLAP	K.YYGTK[242.12]DPVPGYSVPAAEHSTITAWGK.D	P43490	NAMPT	Nicotinamide phosphoribosyltransferase	0.9996
GLAP	K.YGK[242.12]SLYYYIQQDTK.G	P07355	ANXA2	Annexin A2	0.993
GLAP	K.EVHK[242.12]QVVESAYEVIK.L	P00338	LDHA	L-lactate dehydrogenase A chain	0.9392
GLAP	R.K[242.12]DGNASGTTLLEALDC[160.03]ILPPTRPTDKPLR.L	Q5VTE0	EF1A3	Putative elongation factor 1-alpha-like 3	1
GLAP	D.GK[242.12]AIPDLTAPVAAVQAAVSNLVRVGKE.T	P18206	VINC	Vinculin	1
GLAP	E.VK[242.12]K[242.12]EVAFFNNFLTDAKRPALPE.L	Q9BXP5	SRRT	Serrate RNA effector molecule homolog	1
GLAP	K.VEAILEK[242.12]VQGLEQAVDNFEGK.K	O95817	BAG3	BAG family molecular chaperone regulator 3	0.9914
GLAP	R.K[242.12]LEGDSTDLSDQIAELQAQIAELK.M	P35579	MYH9	Myosin-9	0.999
GLAP	K.NIEDVIAQGIGK[242.12]LASVPAGGAVAVSAAPGSAAPAAGSAPAAAEEK.K	P05387	RLA2	60S acidic ribosomal protein P2	1
GLAP	R.TEK[242.12]LLETIDQLYLEYAK.R	P12814	ACTN1	Alpha-actinin-1	0.9739
GLAP	R.DLTDYLMK[242.12]ILTER.G	P68032	ACTC	Actin, alpha cardiac muscle 1	0.9699
GLAP	K.GASDK[242.12]C[160.03]SC[160.03]C[160.03]A.-	P02795	MT2	Metallothionein-2	1
GLAP	R.TVK[242.12]EEAEKPER.E	P14314	GLU2B	Glucosidase 2 subunit beta	0.9973
GLAP	R.LK[242.12]QSAEEQAQAR.A	Q15149	PLEC	Plectin	0.9478
GLAP	K.VVK[242.12]QASEGPLK.G	P04406	G3P	Glyceraldehyde-3-phosphate dehydrogenase	0.986
GLAP	R.HQGVM[147.04]VGM[147.04]GQK[242.12]DSYVGDEAQSK.R	P68032	ACTC	Actin, alpha cardiac muscle 1	0.9996
GLAP	K.IFVGGIK[242.12]EDTEEHHLR.D	Q32P51	RA1L2	Heterogeneous nuclear ribonucleoprotein A1-like 2	1
GLAP	K.EAALSTALSEK[242.12]R.T	P02545	LMNA	Prelamin-A \ C	0.9993
GLAP	K.AQAYQTGK[242.12]DISTNYYASQK.K	P14625	ENPL	Endoplasmin	0.9142
GLAP	R.MQK[242.12]EITALAPSTMK.L	P68032	ACTC	Actin, alpha cardiac muscle 1	0.9602
GLAP	K.AAAGK[242.12]AELELELGR.L	Q15149	PLEC	Plectin	0.9305
GLAP	K.ELLHK[242.12]ELPSFVGEK.V	P29034	S10A2	Protein S100-A2	0.9995

GLAP: glyceraldehyde-derived pyrinidium compound
MG-H1: methylglyoxal-derived AGE or N^{δ}-(5-hydro-5-methyl-4-imidazolon-2-yl)ornithine
ArgP: Argpyrimidine

Table S1. Peptides list of HPDE having AGEs modifications (continued)

AGEs	Peptide	Accession number	Protein	Protein description	Probability
GLAP	R.LIGDAAK[242.12]NQVAMNPTNTVFDAK.R	P11142	HSP7C	Heat shock cognate 71 kDa protein	0.9999
GLAP	R.TLSDYNIQK[242.12]ESTLHLVLR.L	P62987	RL40	Ubiquitin-60S ribosomal protein L40	0.997
GLAP	K.K[242.12]GIVDQSQQAYQEAFEISK.K	P63104	1433Z	14-3-3 protein zeta \ delta	1
GLAP	K.LK[242.12]GEMMDLQHGSLFLR.T	P00338	LDHA	L-lactate dehydrogenase A chain	1
GLAP	K.VVLAYEPVWAIGTGK[242.12]TATPQQAQEVHEK.L	P60174	TPIS	Triosephosphate isomerase	1
GLAP	K.QNIK[242.12]LLDLKPYK.V	O60942	MCE1	mRNA-capping enzyme	0.9972
GLAP	K.TQLEELEDELQATEDAK[242.12]LR.L	P35749	MYH11	Myosin-11	0.98
GLAP	K.ASMK[242.12]GLGTDEDSLIEIIC[160.03]SR.T	P07355	ANXA2	Annexin A2	0.9642
GLAP	R.GVLK[242.12]VFLENVIR.D	P62805	H4	Histone H4	0.9997
GLAP	E.VRGFPTIYFSPANKK[242.12]LNPKKYE.G	P30101	PDIA3	Protein disulfide-isomerase A3	1
GLAP	E.KDIISDTSGDFRKLMVALAK[242.12]GRRAE.D	P07355	ANXA2	Annexin A2	1
GLAP	K.HALK[242.12]GAGTNEK.V	P08758	ANXA5	Annexin A5	0.9999
GLAP	R.GIGTDEK[242.12]MLISILTER.S	P12429	ANXA3	Annexin A3	0.9767
GLAP	K.FISDK[242.12]DASIVGFFDDSFSEAHSEFLK.A	P30101	PDIA3	Protein disulfide-isomerase A3	1
GLAP	R.IVEEANK[242.12]AFEYNMQIFNELDQAGSTLAR.E	P30519	HMOX2	Heme oxygenase 2	1
GLAP	R.DLLDDLK[242.12]SELTGK.F	P08758	ANXA5	Annexin A5	0.9886
GLAP	K.LPEDPLLSGLLDSPALK[242.12]AC[160.03]LDTAVENM[147.04]PSLK[242.12].M	P49327	FAS	Fatty acid synthase	0.9986
GLAP	R.ELAK[242.12]VEEEIQTLSQVLAAK.E	P55327	TPD52	Tumor protein D52	1
GLAP	K.GLK[242.12]NVFDEAILAALEPPEPK.K	P60953	CDC42	Cell division control protein 42 homolog	0.9949
GLAP	K.LC[160.03]YVALDFEQEMATAASSSSLEK[242.12]SYELPDGQVITIGNER.F	Q6S8J3	POTEE	POTE ankyrin domain family member E	1
GLAP	K.IQASTMAFK[242.12]QMEQISQFLQAAER.Y	P37802	TAGL2	Transgelin-2	1
GLAP	R.SIPAYLAETLYYAMK[242.12]GAGTDDHTLIR.V	P08758	ANXA5	Annexin A5	1
GLAP	K.EGLPVALDK[242.12]HILGFDTGDAVLNEAAQILR.L	Q9Y224	RTRAF	RNA transcription, translation and transport factor protein	1
GLAP	R.EKVETELQGVC[160.03]DTVLGLLDSHLIK[242.12]EAGDAESR.V	P31947	1433S	14-3-3 protein sigma	1
GLAP	K.GLYGIK[242.12]DDVFLSVPC[160.03]ILGQNGISDLVK.V	P00338	LDHA	L-lactate dehydrogenase A chain	0.9843
GLAP	R.QITDNIFLTTAEVIAQQVSDK[242.12]HLEEGR.L	P48163	MAOX	NADP-dependent malic enzyme	1
GLAP	R.GIVK[242.12]HLEGLSEEAIMELNLPTGIPIVYELDK.N	P18669	PGAM1	Phosphoglycerate mutase 1	1
GLAP	R.ELQTK[242.12]INEAIVAVQAIIADPK.T	Q9Y224	RTRAF	RNA transcription, translation and transport factor protein	0.9999
GLAP	K.VETELQGVC[160.03]DTVLGLLDSHLIK[242.12]EAGDAESR.V	P31947	1433S	14-3-3 protein sigma	1
GLAP	K.AC[160.03]SLAK[242.12]TAFDEAIAELDTLNEESYK.D	P31946	1433B	14-3-3 protein beta \ alpha	0.9996
GLAP	K.EVK[242.12]GDLENAFLNLVQC[160.03]IQNKPLYFADR.L	P07355	ANXA2	Annexin A2	0.9979
GLAP	R.ALDLFSDNAPPPELLEIINEDIAK[242.12]R.T	Q15084	PDIA6	Protein disulfide-isomerase A6	0.9992
GLAP	R.NIK[242.12]TIAEC[160.03]LADELINAAK.G	P46782	RS5	40S ribosomal protein S5	1
GLAP	R.EK[242.12]VETELQGVC[160.03]DTVLGLLDSHLIK.E	P31947	1433S	14-3-3 protein sigma	1
GLAP	R.KYSVWIGGSILASLSTFQQMWISK[242.12]QEYDESGPSIVHR.K	P60709	ACTB	Actin, cytoplasmic 1	1
GLAP	K.DASIVGFFDDSFSEAHSEFLK[242.12]AASNLR.D	P30101	PDIA3	Protein disulfide-isomerase A3	1
GLAP	K.TTFDEAMADLHTLSEDSYK[242.12]DSTLIMQLLR.D	P31947	1433S	14-3-3 protein sigma	1

GLAP: glyceraldehyde-derived pyrinidium compound

MG-H1: methylglyoxal-derived AGE or N^{δ}-(5-hydro-5-methyl-4-imidazolon-2-yl)ornithine

ArgP: Argpyrimidine

Table S1 . Peptides list of HPDE having AGEs modifications (continued)

AGEs	Peptide	Accession number	Protein	Protein description	Probability
GLAP	R.K[242.12]AMEAVAAQGK.A	P15259	PGAM2	Phosphoglycerate mutase 2	0.9999
GLAP	K.K[242.12]AQQELEEQTR.R	P26038	MOES	Moesin	0.9937
GLAP	K.SNEEGSEEK[242.12]GPEVR.E	P31947	1433S	14-3-3 protein sigma	0.9989
GLAP	K.LPK[242.12]ADIDVSGPK.V	Q09666	AHNK	Neuroblast differentiation-associated protein AHNAK	0.9884
GLAP	R.VLSSIEQK[242.12]SNEEGSEEK.G	P31947	1433S	14-3-3 protein sigma	0.9989
GLAP	R.HQGVMVGMGQK[242.12]DSYVGDEAQSK.R	P68032	ACTC	Actin, alpha cardiac muscle 1	1
GLAP	R.GLK[242.12]YQEGGVESAFHK.T	P40121	CAPG	Macrophage-capping protein	0.9957
GLAP	K.AVEHINK[242.12]TIAPALVSK.K	P06733	ENOA	Alpha-enolase	1
GLAP	R.LLC[160.03]GK[242.12]ETMVTSTTEPSR.C	P14314	GLU2B	Glucosidase 2 subunit beta	0.9958
GLAP	K.SQGK[242.12]VLQATVVAVGSGSK.G	P61604	CH10	10 kDa heat shock protein, mitochondrial	0.9973
GLAP	K.K[242.12]HLEINPDHPIVETLR.Q	P08238	HS90B	Heat shock protein HSP 90-beta	1
GLAP	R.LIFAGK[242.12]QLEDGR.T	P62987	RL40	Ubiquitin-60S ribosomal protein L40	0.9892
GLAP	R.FSGWYDADLSPAGHEEAK[242.12]R.G	P18669	PGAM1	Phosphoglycerate mutase 1	1
GLAP	R.K[242.12]LDELYGTWR.K	P36578	RL4	60S ribosomal protein L4	0.9997
GLAP	K.SSK[242.12]GGPGSAVSPYPTFNPSSDVAALHK.A	P04083	ANXA1	Annexin A1	1
GLAP	K.SIQEIQELDK[242.12]DDESLR.K	P52565	GDIR1	Rho GDP-dissociation inhibitor 1	0.9993
GLAP	R.LLLPGELAK[242.12]HAVSEGTK.A	P58876	H2B1D	Histone H2B type 1-D	0.9981
GLAP	K.TLTGK[242.12]EIEIDIEPTDK.V	Q15843	NEDD8	NEDD8	0.8982
GLAP	K.K[242.12]GVNLPGAAVDLPAVSEK.D	P14618	KPYM	Pyruvate kinase PKM	1
GLAP	R.ASLSK[242.12]LGDVYVNDAFGTAHR.A	P07205	PGK2	Phosphoglycerate kinase 2	0.9995
GLAP	K.EAFSLFDK[242.12]DGDGTITTK.E	P0DP25	CALM3	Calmodulin-3	0.9644
GLAP	K.K[242.12]C[160.03]LELFTELAEDKENYK.K	P07900	HS90A	Heat shock protein HSP 90-alpha	0.9861
GLAP	K.FTASAGIQVVGDDLTVTNPK[242.12]R.L	P06733	ENOA	Alpha-enolase	0.9968
GLAP	R.YDDMAAC[160.03]MK[242.12]SVTEQGAELSNEER.N	P63104	1433Z	14-3-3 protein zeta \ delta	1
GLAP	R.K[242.12]DLYANTVLSGGTTMYPGIADR.M	P60709	ACTB	Actin, cytoplasmic 1	1
GLAP	K.VYEGERPLTK[242.12]DNHLLGTFDLTGIPPAPR.G	P11021	BIP	Endoplasmic reticulum chaperone BiP	1
GLAP	R.YDDMATC[160.03]MK[242.12]AVTEQGAELSNEER.N	P27348	1433T	14-3-3 protein theta	1
GLAP	R.VLMEK[242.12]EFPGFLENQK.D	P60903	S10AA	Protein S100-A10	0.9967
GLAP	K.GIVDQSQQAYQEAFEISK[242.12]K.E	P63104	1433Z	14-3-3 protein zeta \ delta	0.8905
GLAP	R.EK[242.12]YGIVDYMIEQSGPPSK.E	P13667	PDIA4	Protein disulfide-isomerase A4	0.9999
GLAP	K.K[242.12]TQEQLALEMAELTAR.L	P26038	MOES	Moesin	1
GLAP	K.NEK[242.12]VIEHIMEDLDTNADK.Q	P06702	S10A9	Protein S100-A9	0.9963
GLAP	K.SLTNDWEDHLAVK[242.12]HFSVEGQLEFR.A	P08238	HS90B	Heat shock protein HSP 90-beta	1
GLAP	R.YDDMAAAMK[242.12]NVTELNEPLSNEER.N	P61981	1433G	14-3-3 protein gamma	1
GLAP	R.AELTK[242.12]VEEEIVTLR.Q	O43399	TPD54	Tumor protein D54	1
GLAP	K.VGNIEIK[242.12]DLMVGDEASELR.S	P61160	ARP2	Actin-related protein 2	0.9987
GLAP	R.LPLQDVYK[242.12]IGGIGTVPVGR.V	Q5VTE0	EF1A3	Putative elongation factor 1-alpha-like 3	1
GLAP	K.HSQFIGYPITLYLEK[242.12]ER.E	P08238	HS90B	Heat shock protein HSP 90-beta	0.9988
GLAP	R.AK[242.12]FEELNMDLFR.S	P11021	BIP	Endoplasmic reticulum chaperone BiP	0.9999

GLAP: glyceraldehyde-derived pyrinidium compound
MG-H1: methylglyoxal-derived AGE or N^{δ}-(5-hydro-5-methyl-4-imidazolon-2-yl)ornithine
ArgP: Argpyrimidine

Table S1 . Peptides list of HPDE having AGEs modifications (continued)

AGEs	Peptide	Accession number	Protein	Protein description	Probability
GLAP	K.YGK[242.12]DATNVGDEGGFAPNILENKEGLELLK.T	P06733	ENOA	Alpha-enolase	1
GLAP	K.VVEVGSK[242.12]IYVDDGLISLQVK.Q	P14618	KPYM	Pyruvate kinase PKM	0.9977
GLAP	R.QAVDQIK[242.12]SQEQLAAELAEYTAK.L	P15311	EZRI	Ezrin	1
GLAP	R.VETGVLK[242.12]PGMVVTFAPVNVTTEVK.S	Q5VTE0	EF1A3	Putative elongation factor 1-alpha-like 3	1
GLAP	R.HVGDLGNVTADK[242.12]DGVADVSIEDSVISLSGDHC[160.03]IIGR.T	P00441	SODC	Superoxide dismutase [Cu-Zn]	1
GLAP	R.GILTLK[242.12]YPIEHGIITNWDDMEK.L	P68032	ACTC	Actin, alpha cardiac muscle 1	0.9866
GLAP	K.YLDIPK[242.12]MLDAEDIVNTARPDEK.A	O43707	ACTN4	Alpha-actinin-4	0.9997
GLAP	R.ADGYVLEGK[242.12]ELEFYLR.K	P62241	RS8	40S ribosomal protein S8	0.9454
GLAP	K.GTDVPK[242.12]WISIMTER.S	P07355	ANXA2	Annexin A2	1
GLAP	K.TFVNITPAEVGVLVGK[242.12]DR.S	P07737	PROF1	Profilin-1	1
GLAP	K.MYDAAK[242.12]LLYNNVSNFGR.L	Q00610	CLH1	Clathrin heavy chain 1	0.9885
GLAP	R.K[242.12]HGLEVIYMIEPIDEYC[160.03]VQQLK.E	P07900	HS90A	Heat shock protein HSP 90-alpha	1
GLAP	R.QFASQANVVGPWIQTK[242.12]MEEIGR.L	O43707	ACTN4	Alpha-actinin-4	0.9286
GLAP	K.YPIEHGIITNWDDMEK[242.12]IWHHTFYNELR.V	P68032	ACTC	Actin, alpha cardiac muscle 1	0.9999
GLAP	R.LIALLEVLSQK[242.12]R.M	Q14315	FLNC	Filamin-C	0.9993
GLAP	K.VGK[242.12]DELFALEQSC[160.03]AQVVLQAANER.N	Q16658	FSCN1	Fascin	1
GLAP	R.AAMK[242.12]GLGTDEDTLIEILASR.T	P04083	ANXA1	Annexin A1	0.9999
GLAP	K.LK[242.12]SMEAEMIQLQEELAAAER.A	P35579	MYH9	Myosin-9	0.974
GLAP	K.QFGAQANVIGPWIQTK[242.12]MEEIGR.L	P12814	ACTN1	Alpha-actinin-1	0.9997
GLAP	R.K[242.12]LPIDVTEGEVISLGLPFGK.V	P26599	PTBP1	Polypyrimidine tract-binding protein 1	1
GLAP	R.TLGLYGK[242.12]DQQEAALVDMVNDGVEDLR.C	P09211	GSTP1	Glutathione S-transferase P	1
GLAP	K.AMK[242.12]GLGTDEESILTLLTSR.S	P08758	ANXA5	Annexin A5	1
GLAP	K.EK[242.12]LC[160.03]YVALDFEQEMATAASSSSLEK.S	Q6S8J3	POTEE	POTE ankyrin domain family member E	1
GLAP	K.HSQFIGYPITLFVEK[242.12]K[242.12].R	Q58FG0	HS905	Putative heat shock protein HSP 90-alpha A5	1
GLAP	K.SFIK[242.12]DYPVVSIEDPFDQDDWGAWQK.F	P06733	ENOA	Alpha-enolase	1
GLAP	R.K[242.12]PLVIIAEDVDGEALSTLVLNR.L	P10809	CH60	60 kDa heat shock protein, mitochondrial	1
GLAP	R.AK[242.12]FYPEDVSEELIQDITQR.L	P26038	MOES	Moesin	0.9996
GLAP	K.VSHVSTGGGASLELLEGK[242.12]VLPGVDALSNI.-	P00558	PGK1	Phosphoglycerate kinase 1	1
GLAP	R.AIEK[242.12]LAVEALSSLDGDLAGR.Y	P12277	KCRB	Creatine kinase B-type	0.9996
GLAP	E.DGVTPYMIFFK[242.12]DGLE.M	P13693	TPT1	Translationally-controlled tumor protein	0.9943
GLAP	E.YWTLIGGITGPIAK[242.12]LIHE.Q	Q96FQ6	S10AG	Protein S100-A16	0.9899
GLAP	D.LAGRDLTDYLMK[242.12]ILTE.R	P68032	ACTC	Actin, alpha cardiac muscle 1	0.9745
GLAP	E.LRDNDKTRYMGKGVSK[242.12]AVE.H	P06733	ENOA	Alpha-enolase	1
GLAP	E.KQDK[242.12]IYFMAGSSRKE.A	P14625	ENPL	Endoplasmin	0.9785
GLAP	E.YVK[242.12]RALANSLAC[160.03]QGKYTPSGQAGAAASE.S	P04075	ALDOA	Fructose-bisphosphate aldolase A	0.9991
GLAP	E.GKRVLIAAHGNSLRGIVK[242.12]HLE.G	P18669	PGAM1	Phosphoglycerate mutase 1	1
GLAP	E.RIMKAQAYQTGKDISTNYYASQKK[242.12]TFE.L	P14625	ENPL	Endoplasmin	1
GLAP	E.HINK[242.12]TIAPALVSKKLNVTE.Q	P06733	ENOA	Alpha-enolase	1
GLAP	E.VRGFPTIYFSPANK[242.12]KLNPKKYE.G	P30101	PDIA3	Protein disulfide-isomerase A3	1
GLAP	D.LYANTVLSGGTTMYPGIADRMQK[242.12]E.L	P60709	ACTB	Actin, cytoplasmic 1	0.9945

GLAP: glyceraldehyde-derived pyrinidium compound
MG-H1: methylglyoxal-derived AGE or N$^{\delta}$-(5-hydro-5-methyl-4-imidazolon-2-yl)ornithine
ArgP: Argpyrimidine

Table S1. Peptides list of HPDE having AGEs modifications (continued)

AGEs	Peptide	Accession number	Protein	Protein description	Probability
GLAP	E.KYDNSLKIISNASC[160.03]TTNC[160.03]LAPLAK[242.12]VIHD.N	P04406	G3P	Glyceraldehyde-3-phosphate dehydrogenase	1
GLAP	E.QALAVLVTTFHK[242.12]YSC[160.03]QE.G	P29034	S10A2	Protein S100-A2	1
GLAP	E.DVIAQGIGK[242.12]LASVPAGGAVAVSAAPGSAAPAAGSAPAAAEE.K	P05387	RLA2	60S acidic ribosomal protein P2	0.9977
GLAP	D.AGTIAGLNVMRIINEPTAAAIAYGLDK[242.12]RE.G	P11021	BIP	Endoplasmic reticulum chaperone BiP	0.9999
MG-H1	K.STNC[160.03]VVDSR[213.11].M	P25092	GUC2C	Heat-stable enterotoxin receptor	0.9987
MG-H1	K.DSYVGDEAQSKR[213.11].G	P68032	ACTC	Actin, alpha cardiac muscle 1	0.9925
MG-H1	R.TTGIVMDSGDGVTHTVPIYEGYALPHAILR[213.11].L	P60709	ACTB	Actin, cytoplasmic 1	1
MG-H1	D.KTR[213.11]YMGKGVSKAVE.H	P06733	ENOA	Alpha-enolase	1
MG-H1	R.FC[160.03]LDSAR[213.11].Q	P78312	F193A	Protein FAM193A	0.9793
MG-H1	K.GAAAAAAASGAAGGGGGGAGAGAPGGGR[213.11].L	Q68E01	INT3	Integrator complex subunit 3	0.9959
MG-H1	K.R[213.11]ALSSQHQAR.L	P11021	BIP	Endoplasmic reticulum chaperone BiP	0.9995
MG-H1	R.APAHAAR[213.11]AK.E	Q4ZHG4	FNDC1	Fibronectin type III domain-containing protein 1	0.9878
MG-H1	K.C[160.03]VSKR[213.11]IANLQTDLSDGLR.L	P21333	FLNA	Filamin-A	0.9823
MG-H1	R.AAGR[213.11]GVPAGVPIPQAPAGLAGPVR.G	P63162	RSMN	Small nuclear ribonucleoprotein-associated protein N	0.9227
MG-H1	E.KDIISDTSGDFRKLMVALAKGR[213.11]R[213.11]AE.D	P07355	ANXA2	Annexin A2	1
ArgP	K.IGNC[160.03]PFSQR[241.13]LFMVLWLKGVTFNVTTVDTK.R	O00299	CLIC1	Chloride intracellular channel protein 1	1
ArgP	R.C[160.03]EC[160.03]R[241.13]SGYEFADDR.H	Q14112	NID2	Nidogen-2	0.9995
ArgP	K.R[241.13]VIISAPSADAPMFVMGVNHEK.Y	P04406	G3P	Glyceraldehyde-3-phosphate dehydrogenase	1
ArgP	R.TRAC[160.03]DQPPPQGLGDYC[160.03]EGPR[241.13].A	A2VEC9	SSPO	SCO-spondin	0.958
ArgP	D.LFR[241.13]AIFASSSDEKSSSSE.D	Q9BRR8	GPTC1	G patch domain-containing protein 1	1
ArgP	E.LALVR[241.13]FVVED.Y	Q8N3E9	PLCD3	1-phosphatidylinositol 4,5-bisphosphate phosphodiesterase delta-3	0.9869
ArgP	E.KGLASLKSEM[147.04]R[241.13]EVEGE.L	Q13753	LAMC2	Laminin subunit gamma-2	0.9726
ArgP	K.EM[147.04]ERM[147.04]VSIIHR[241.13]KFSAIQM[147.04]QLK.Q	P40425	PBX2	Pre-B-cell leukemia transcription factor 2	0.9999
ArgP	E.NSPNSFPR[241.13]R[241.13]E.R	Q9P260	RELCH	RAB11-binding protein RELCH	0.9882
ArgP	E.KELSR[241.13]IEEALMDPGR[213.11]QPE.S	Q14690	RRP5	Protein RRP5 homolog	0.9445
ArgP	E.TALITR[241.13]PGAPSRRKE.V	O94760	DDAH1	N(G),N(G)-dimethylarginine dimethylaminohydrolase 1	0.9894
GLAP/ArgP	K.R[241.13]K[242.12]TVTAMDVVYALKR.Q	P62805	H4	Histone H4	1
GLAP/MG-H1	E.VVKKHSQFIGYPITLYLEK[242.12]ER[213.11]E.K	P08238	HS90B	Heat shock protein HSP 90-beta	0.9768
GLAP/MG-H1	D.GR[213.11]ISITRVTADLSLAKR[213.11]SVLNNPGK[242.12]RTIIE.R	P54284	CACB3	Voltage-dependent L-type calcium channel subunit beta-3	1
MG-H1/ArgP	D.KADLSAR[213.11]VTELGLAVKR[241.13]LEKQNLE.K	Q5TZA2	CROCC	Rootletin	1
MG-H1/ArgP	E.LVM[147.04]EILSDLLKR[213.11]LPLTVEKE.E	Q0VDD8	DYH14	Dynein heavy chain 14, axonemal	0.9998

GLAP: glyceraldehyde-derived pyrinidium compound
MG-H1: methylglyoxal-derived AGE or N^{δ}-(5-hydro-5-methyl-4-imidazolon-2-yl)ornithine
ArgP: Argpyrimidine

Table S2. Peptides list of PANC-1 having AGEs modifications

AGEs	Peptide	Accession number	Protein	Protein description	Probability
GLAP	K.K[242.12]GK[242.12]QEEEKPGEEK.T	O60508	PRP17	Pre-mRNA-processing factor 17	0.9913
GLAP	K.LPK[242.12]ADIDVSGPK.V	Q09666	AHNK	Neuroblast differentiation-associated protein AHNAK	0.999
GLAP	K.LDPK[242.12]IAVAAQNC[160.03]YK.V	P60174	TPIS	Triosephosphate isomerase	1
GLAP	R.SLDMDSIIAEVK[242.12]AQYEDIANR.S	P05787	K2C8	Keratin, type II cytoskeletal 8	0.9979
GLAP	R.LTELC[160.03]EEVK[242.12].K	Q9P2D6	F135A	Protein FAM135A	0.992
GLAP	K.EAFNMIDQNRDGFIDK[242.12]EDLHDMLASLGK.N	P24844	MYL9	Myosin regulatory light polypeptide 9	1
GLAP	K.AGYTDK[242.12]VVIGMDVAASEFFR.S	P06733	ENOA	Alpha-enolase	1
GLAP	E.KNKLGAKIIK[242.12]AEM[147.04]M[147.04]GNM[147.04]E.L	Q2TBE0	C19L2	CWF19-like protein 2	1
GLAP	K.AC[160.03]SLAK[242.12]TAFDEAIAELDTLSEESYK.D	P63104	1433Z	14-3-3 protein zeta\delta	1
GLAP	K.ELASALK[242.12]SALSGHLETVILGLLK.T	P07355	ANXA2	Annexin A2	0.9996
GLAP	K.DSYVGDEAQSK[242.12]R.G	P68032	ACTC	Actin, alpha cardiac muscle 1	0.9995
GLAP	R.HQGVMVGMGQK[242.12]DSYVGDEAQSK.R	P68032	ACTC	Actin, alpha cardiac muscle 1	1
GLAP	K.K[242.12]HLEINPDHPIVETLR.Q	P08238	HS90B	Heat shock protein HSP 90-beta	1
GLAP	R.MQK[242.12]EITALAPSTMK.L	P68032	ACTC	Actin, alpha cardiac muscle 1	0.9932
GLAP	R.TLSDYNIQK[242.12]ESTLHLVLR.L	P62987	RL40	Ubiquitin-60S ribosomal protein L40	1
GLAP	K.TLTGK[242.12]TITLEVEPSDTIENVK.A	P62987	RL40	Ubiquitin-60S ribosomal protein L40	0.9999
GLAP	R.K[242.12]DLYANTVLSGGTTMYPGIADR.M	P60709	ACTB	Actin, cytoplasmic 1	1
GLAP	R.YDDMATC[160.03]MK[242.12]AVTEQGAELSNEER.N	P27348	1433T	14-3-3 protein theta	1
GLAP	K.DANAK[242.12]LSELEAALQR.A	P05787	K2C8	Keratin, type II cytoskeletal 8	0.9999
GLAP	K.AGYTDK[242.12]VVIGM[147.04]DVAASEFFR.S	P06733	ENOA	Alpha-enolase	1
GLAP	R.K[242.12]DGNASGTTLLEALDC[160.03]ILPPTRPTDKPLR.L	Q5VTE0	EF1A3	Putative elongation factor 1-alpha-like 3	1
GLAP	R.QFASQANVVGPWIQTK[242.12]MEEIGR.L	O43707	ACTN4	Alpha-actinin-4	1
GLAP	K.LQHLENELTHDIITK[242.12]FLENEDR.R	P01009	A1AT	Alpha-1-antitrypsin	1
GLAP	K.LK[242.12]LEAELGNMQGLVEDFK.N	P05787	K2C8	Keratin, type II cytoskeletal 8	0.9999
GLAP	R.K[242.12]YSNEDTLSVALPYFWEHFDK.D	P26641	EF1G	Elongation factor 1-gamma	1
GLAP	K.SFIK[242.12]DYPVVSIEDPFDQDDWGAWQK.F	P06733	ENOA	Alpha-enolase	1
GLAP	D.K[242.12]FASFIDKVRFLE.Q	Q7RTS7	K2C74	Keratin, type II cytoskeletal 74	1
MG-H1	K.GYLIFVVR[213.11].F	Q96P20	NLRP3	NACHT, LRR and PYD domains-containing protein 3	0.9139
MG-H1	E.NYGPR[213.11]AE.V	Q5JVS0	HABP4	Intracellular hyaluronan-binding protein 4	0.9418
MG-H1	K.AFAKPSQLER[213.11].H	Q9UL36	ZN236	Zinc finger protein 236	0.9966
MG-H1	R.VLHWDLR[213.11].G	Q9BRK3	MXRA8	Matrix remodeling-associated protein 8	0.9037
MG-H1	E.KSSLGAVAR[213.11]EEAKPE.S	Q96D09	GASP2	G-protein coupled receptor-associated sorting protein 2	0.9926
MG-H1	K.AVPFPPTHR[213.11].L	P16298	PP2BB	Serine\threonine-protein phosphatase 2B catalytic subunit beta isoform	0.999
ArgP	R.R[241.13]EDM[147.04]ASVLEM[147.04]SVQFLR.L	Q5JUK2	SOLH1	Spermatogenesis- and oogenesis-specific basic helix-loop-helix-containing protein 1	0.9987
ArgP	K.R[241.13]ADVGEFF.-	O00458	IFRD1	Interferon-related developmental regulator 1	0.956
GLAP/ArgP	R.KLAEQFPR[241.13]QVLDSK[242.12]APK[242.12]PEDIDEEDDDVPDLVENFDEASK.N	Q96K17	BT3L4	Transcription factor BTF3 homolog 4	0.995
GLAP/ArgP	K.K[242.12]DVDEAYM[147.04]NKVELESR[241.13]LEGLTDEINFLR.Q	P05787	K2C8	Keratin, type II cytoskeletal 8	0.9966
GLAP/MG-H1/ArgP	D.KREPYEGLRPQDLR[241.13]R[213.11]LK[242.12]DMLIVE.T	Q9P2G1	AKIB1	Ankyrin repeat and IBR domain-containing protein 1	0.9999

GLAP: glyceraldehyde-derived pyrinidium compound
MG-H1: methylglyoxal-derived AGE or N^{δ}- (5 hydro 5-methyl-4-imidazolon-2-yl)ornithine
ArgP: Argpyrimidine

Table S3. The 11 shared proteins between PANC-1 and HPDE

Protein Abbreviation	Protein ID	Protein Name
TPI1	9606.ENSP00000229270	Triosephosphate isomerase 1
ENO1	9606.ENSP00000234590	Alpha-enolase
ACTN4	9606.ENSP00000252699	Alpha-actinin-4
ACTC1	9606.ENSP00000290378	Actin, alpha cardiac muscle 1
ANXA2	9606.ENSP00000346032	Annexin A2
ACTB	9606.ENSP00000349960	Actin, cytoplasmic 1
CDC40	9606.ENSP00000357928	Pre-mRNA-processing factor 17
HSP90AB1	9606.ENSP00000360609	Heat shock protein HSP 90-beta
AHNAK	9606.ENSP00000367263	Neuroblast differentiation-associated protein AHNAK
YWHAZ	9606.ENSP00000379287	14-3-3 protein zeta/delta
UBA52	9606.ENSP00000388107	Ubiquitin-60S ribosomal protein L40

Table S4. Peptides having multiple AGEs in HPDE treated with glyceraldehyde

Glyceraldehyde concentration	Modified peptide	Protein Abbr.	Protein Name
1 mM	R.SVFLGK[242.12]IK[242.12]DAFDRNPELQNLLLDDFFK.S	PGD	6-phosphogluconate dehydrogenase, decarboxylating
	K.R[241.13]K[242.12]TVTAMDVVYALKR.Q	H4C1	Histone H4
	K.K[242.12]GK[242.12]QEEEKPGEEK.T	CDC40/PRP17	Pre-mRNA-processing factor 17
	D.KADLSAR[213.11]VTELGLAVKR[241.13]LEKQNLE.K	CROCC	Rootletin
2 mM	K.K[242.12]GK[242.12]QEEEKPGEEK.T	CDC40/PRP17	Pre-mRNA-processing factor 17
	E.VVKKHSQFIGYPITLYLEK[242.12]ER[213.11]E.K	HS90B	Heat shock protein HSP 90-beta
4 mM	K.HSQFIGYPITLFVEK[242.12]K[242.12].R	HSP90AA5P	Putative heat shock protein HSP 90-alpha A5
	K.LPEDPLLSGLLDSPALK[242.12]AC[160.03]LDTAVENM[147.04]PSLK[242.12].M	FASN	Fatty acid synthase
	K.K[242.12]GK[242.12]QEEEKPGEEK.T	CDC40/PRP17	Pre-mRNA-processing factor 17
	K.R[241.13]K[242.12]TVTAMDVVYALKR.Q	H4C1	Histone H4
	E.NSPNSFPR[241.13]R[241.13]E.R	RELCH	RAB11-binding protein RELCH
	E.KELSR[241.13]IEEALMDPGR[213.11]QPE.S	RRP5	Protein RRP5 homolog
	D.GR[213.11]ISITRVTADLSLAKR[213.11]SVLNNPGK[242.12]RTIIE.R	CACB3	Voltage-dependent L-type calcium channel subunit beta-3
	E.KDIISDTSGDFRKLMVALAKGR[213.11]R[213.11]AE.D	ANXA2	Annexin A2

Table S5. Peptides having multiple AGEs in PANC-1 treated with glyceraldehyde

Glyceraldehyde Concentration	Modified peptide	Protein Abbr.	Protein Name
1 mM	D.KREPYEGLRPQDLR[241.13]R[213.11]LK[242.12]DMLIVE.T	AKIB1	Ankyrin repeat and IBR domain-containing protein 1
	K.K[242.12]GK[242.12]QEEEKPGEEK.T	CDC40/PRP17	Pre-mRNA-processing factor 17
4 mM	R.KLAEQFPR[241.13]QVLDSK[242.12]APK[242.12]PEDIDEEDDDVPDLVENFDEASK.N	BTF3L4	Transcription factor BTF3 homolog 4
	K.K[242.12]DVDEAYM[147.04]NKVELESR[241.13]LEGLTDEINFLR.Q	KRT8	Keratin, type II cytoskeletal 8
	K.K[242.12]GK[242.12]QEEEKPGEEK.T	CDC40/PRP17	Pre-mRNA-processing factor 17

Table S6. Glycated proteins in HPDE cells

Protein Abbr.	Protein name	Protein Abbr.	Protein name
ACTB	Actin, cytoplasmic 1	LDHA	Lactate dehydrogenase A
ACTC1	Actin, alpha cardiac muscle 1	LMNA	Prelamin-A/C
ACTN1	Alpha-actinin-1	ME1	Malic enzyme 1
ACTN4	Alpha-actinin-4	MSN	Moesin
ACTR2	Actin-related protein 2	MT2A	Metallothionein-2
AHNAK	Neuroblast differentiation-associated protein AHNAK	MYH11	Myosin-11
ALDOA	Fructose-bisphosphate aldolase A	MYH9	Myosin-9
ANXA1	Annexin A1	NAMPT	Nicotinamide phosphoribosyltransferase
ANXA2	Annexin A2	NEDD8	NEDD8
ANXA3	Annexin A3	NID2	Nidogen-2
ANXA5	Annexin A5	PBX2	Pre-B-cell leukemia transcription factor 2
ARHGDIA	Rho GDP-dissociation inhibitor 1	PDCD11	Protein RRP5 homolog
BAG3	BAG family molecular chaperone regulator 3	PDIA3	Protein disulfide-isomerase A3
C14orf166	RNA transcription, translation and transport factor protein	PDIA4	Protein disulfide-isomerase A4
CACNB3	Voltage-dependent L-type calcium channel subunit beta-3	PDIA6	Protein disulfide-isomerase A6
CALM3	Calmodulin 3 (phosphorylase kinase, delta)	PFN1	Profilin-1
CAPG	Macrophage-capping protein	PGAM1	Phosphoglycerate mutase 1
CDC40	Pre-mRNA-processing factor 17	PGAM2	Phosphoglycerate mutase 2
CDC42	Cell division control protein 42 homolog	PGD	6-phosphogluconate dehydrogenase, decarboxylating
CKB	Creatine kinase B-type	PGK1	Phosphoglycerate kinase 1
CLIC1	Chloride intracellular channel protein 1	PGK2	Phosphoglycerate kinase 2
CLTC	Clathrin heavy chain 1	PKM	Pyruvate kinase PKM
CNTN3	Contactin-3	PLCD3	1-phosphatidylinositol 4,5-bisphosphate phosphodiesterase delta-3
CROCC	Rootletin	PLEC	Plectin
DDAH1	N(G),N(G)-dimethylarginine dimethylaminohydrolase 1	POTEE	POTE ankyrin domain family member E
DNAH14	Dynein axonemal heavy chain 14	PRKCSH	Glucosidase 2 subunit beta
ENO1	Alpha-enolase	PTBP1	Polypyrimidine tract-binding protein 1
EZR	Ezrin	RNGTT	mRNA-capping enzyme
FAM193A	Protein FAM193A	RPL4	L ribosomal proteins
FASN	Fatty acid synthase	RPLP2	60S acidic ribosomal protein P2
FLNA	Filamin-A	RPS5	Ribosomal protein S5
FLNC	Filamin-C	RPS8	Ribosomal protein S8
FNDC1	Fibronectin type III domain-containing protein 1	S100A10	Protein S100-A10
FSCN1	Fascin	S100A16	Protein S100-A16
GAPDH	Glyceraldehyde-3-phosphate dehydrogenase	S100A2	Protein S100-A2
GPATCH1	G-patch domain containing 1	S100A9	Protein S100-A9
GSTP1	Glutathione S-transferase P	SFN	14-3-3 protein sigma
GUCY2C	Heat-stable enterotoxin receptor	SNRPN	Small nuclear ribonucleoprotein-associated protein N
HIST1H2BD	Histone H2B type 1-D	SOD1	Superoxide dismutase [Cu-Zn]
HIST4H4	Histone cluster 4, H4	SRRT	Serrate RNA effector molecule homolog
HMOX2	Heme oxygenase 2	TAGLN2	Transgelin-2
HNRNPA1L2	Heterogeneous nuclear ribonucleoprotein A1-like 2	TPD52	Tumor protein D52
HSP90AA1	Heat shock protein HSP 90-alpha	TPD52L2	Tumor protein D52 like 2
HSP90AB1	Heat shock protein HSP 90-beta	TPI1	Triosephosphate isomerase 1
HSP90B1	Endoplasmin	TPT1	Translationally-controlled tumor protein
HSPA5	78 kDa glucose-regulated protein	UBA52	Ubiquitin-60S ribosomal protein L40
HSPA8	Heat shock cognate 71 kDa protein	VCL	Vinculin
HSPD1	60 kDa heat shock protein, mitochondrial	YWHAB	14-3-3 protein beta/alpha
HSPE1	10 kDa heat shock protein, mitochondrial	YWHAE	14-3-3 protein epsilon
INTS3	Integrator complex subunit 3	YWHAG	14-3-3 protein gamma
KIAA1468	LisH domain and HEAT repeat-containing protein KIAA1468	YWHAQ	14-3-3 protein theta
LAMC2	Laminin subunit gamma-2	YWHAZ	14-3-3 protein zeta/delta

Table S7. Glycated proteins in PANC-1 cells

Protein Abbr.	Protein name
ACTB	Actin, cytoplasmic 1
ACTC1	Actin, alpha cardiac muscle 1
ACTN4	Alpha-actinin-4
AHNAK	Neuroblast differentiation-associated protein AHNAK
ANKIB1	Ankyrin repeat and IBR domain-containing protein 1
ANXA2	Annexin A2
BTF3L4	Basic transcription factor 3 like 4
CDC40	Pre-mRNA-processing factor 17
CWF19L2	CWF19-like protein 2
EEF1G	Elongation factor 1-gamma
ENO1	Alpha-enolase
FAM135A	Protein FAM135A
GPRASP2	G-protein coupled receptor-associated sorting protein 2
HABP4	Intracellular hyaluronan-binding protein 4
HSP90AB1	Heat shock protein HSP 90-beta
IFRD1	Interferon-related developmental regulator 1
KRT74	Keratin, type II cytoskeletal 74
KRT8	Keratin, type II cytoskeletal 8
MXRA8	Matrix remodeling-associated protein 8
MYL9	Myosin regulatory light polypeptide 9
NLRP3	NACHT, LRR and PYD domains-containing protein 3
PPP3CB	Serine/threonine-protein phosphatase 2B catalytic subunit beta isoform
SERPINA1	Alpha-1-antitrypsin
SOHLH1	Spermatogenesis- and oogenesis-specific basic helix-loop-helix-containing protein 1
TPI1	Triosephosphate isomerase 1
UBA52	Ubiquitin-60S ribosomal protein L40
YWHAZ	14-3-3 protein zeta/delta
ZNF236	Zinc finger protein 236

Table S8. Glycated proteins in HPDE cells involved in different functions

**Biological Processes**	**Glycated proteins**
glycolytic process	ALDOA,ENO1,GAPDH,LDHA,PGAM1,PGAM2,PGK1,PGK2,PKM,TPI1
oxidation-reduction process	ALDOA,ENO1,FASN,GAPDH,GSTP1,HMOX2,LDHA,ME1,PDIA3,PDIA4,PDIA6,PGAM1,PGAM2,PGD,PGK1,PKM,SOD1,TPI1
catabolic process	ALDOA,ANXA2,CLTC,DDAH1,ENO1,GAPDH,HMOX2,HSP90AA1,HSP90B1,HSPA5,HSPA8,LDHA,NEDD8,PGAM1,PGAM2,PGK1, PGK2,PKM,PLCD3,RPL4,RPLP2,RPS5,RPS8,S100A9,TPI1,UBA52
response to stress	ACTN4,ACTR2,ANXA1,ANXA3,ANXA5,BAG3,CDC42,FLNA,GAPDH,GSTP1,HIST4H4,HMOX2,HSP90AA1,HSP90AB1,HSP90B1,HSPA5, HSPA8,HSPD1,HSPE1,INTS3,LDHA,LMNA,MT2A,PDIA3,PDIA4,PDIA6,PGK1,PKM,S100A9,SFN,SOD1,UBA52,YWHAE,YWHAZ
immune response	ACTR2,ALDOA,ANXA1,ANXA2,ANXA3,GAPDH,GSTP1,HMOX2,HSP90AA1,HSP90AB1,HSPA8,HSPD1,MT2A,PGAM1,PKM,S100A9, VCL
**Molecular Functions**	**Glycated proteins**
S100 protein binding	AHNAK,ANXA2,EZR
oxidoreductase activity	FASN,GAPDH,GSTP1,HMOX2,LDHA,ME1,PDIA3,PDIA4,PDIA6,PGD,PGK1,SOD1
oxidoreductase activity	FASN,GAPDH,GSTP1,HMOX2,LDHA,ME1,PDIA3,PDIA4,PDIA6,PGD,PGK1,SOD1
antioxidant activity	GSTP1,S100A9,SOD1
**KEGG pathway**	**Glycated proteins**
Glycolysis / Gluconeogenesis	ALDOA,ENO1,GAPDH,LDHA,PGAM1,PGAM2,PGK1,PGK2,PKM,TPI1
Carbon metabolism	ALDOA,ENO1,GAPDH,ME1,PGAM1,PGAM2,PGD,PGK1,PGK2,PKM,TPI1
Central carbon metabolism in cancer	LDHA,PGAM1,PGAM2,PKM
Proteoglycans in cancer	ACTB,CDC42,EZR,FLNA,FLNC,MSN
**Reactome Pathway**	**Glycated proteins**
Gluconeogenesis	GAPDH,TPI1,ENO1,PGAM2,PGK2,PGAM1,PGK1,ALDOA
Glycolysis	GAPDH,TPI1,ENO1,PGAM2,PGK2,PGAM1,PGK1,ALDOA
Immune System	VCL,MYH9,MT2A,SOD1,HSP90B1,PDIA3,PKM,HSPA5,HSP90AA1,ANXA2,ACTB,MSN,S100A9,FLNA,PGAM1,HSP90AB1,YWHAB, ANXA1,ACTR2,FSCN1,ALDOA,YWHAZ,GSTP1,CDC42,UBA52,HSPA8,PRKCSH,HMOX2,CLTC
G2/M DNA damage checkpoint	YWHAE,HIST1H2BD,YWHAG,SFN,YWHAB,YWHAQ,YWHAZ
Apoptosis	YWHAE,YWHAG,PLEC,SFN,YWHAB,YWHAQ,YWHAZ,UBA52
TP53 Regulates Metabolic Genes	YWHAE,YWHAG,SFN,YWHAB,YWHAQ,YWHAZ
