# Supplementary material for: Proteomic Investigation of Glyceraldehyde-Derived Intracellular AGEs and Their Potential Influence on Pancreatic Ductal Cells
Source: Cells. 2021 Apr 24;10(5):1005. doi: 10.3390/cells10051005 (PMC8145644; doi:10.3390/cells10051005)
Supplement: Supplementary file 1 [file cells-10-01005-s001.zip › Supp_files_proofread/Supplemeantary figures_proof read.pdf]

Supplementary Figures

Figure S1. Venn-diagram showing number of modified proteins through glyceraldehyde treatment

Figure S2. PANC-1 and MIA PaCa-2 cells treated with glyceraldehyde for 48 hrs in serum free media. (A, D) Bright field images of cells treated for 48 hrs with glyceraldehyde. Scale bar: 200 μm. (B, E) Cell viability determined by MTT assay. (C, F) Cell damage determined by LDH assay. Absorbance or fluorescence values were normalized to control (0 μM). Data represent the mean (n=3)± SE. ****P<0.0001, ***P<0.001, **P< 0.01, *P< 0.05 vs. control (0 mM)

Figure S3. Functional annotations of AGEs modified proteins in glyceraldehyde treated HPDE cells (A) GO analysis-Biological Processes (B) GO analysis-Molecular Functions (C) GO analysis-Cellular components (D) KEGG pathway analysis (E) Reactome Pathway analysis

Figure S4. Functional annotations of AGEs modified proteins in glyceraldehyde treated PANC-1 cells (A) GO analysis-Molecular functions (B) GO analysis-Cellular components (C) KEGG pathway analysis (D) Reactome Pathway analysis

Figure S1

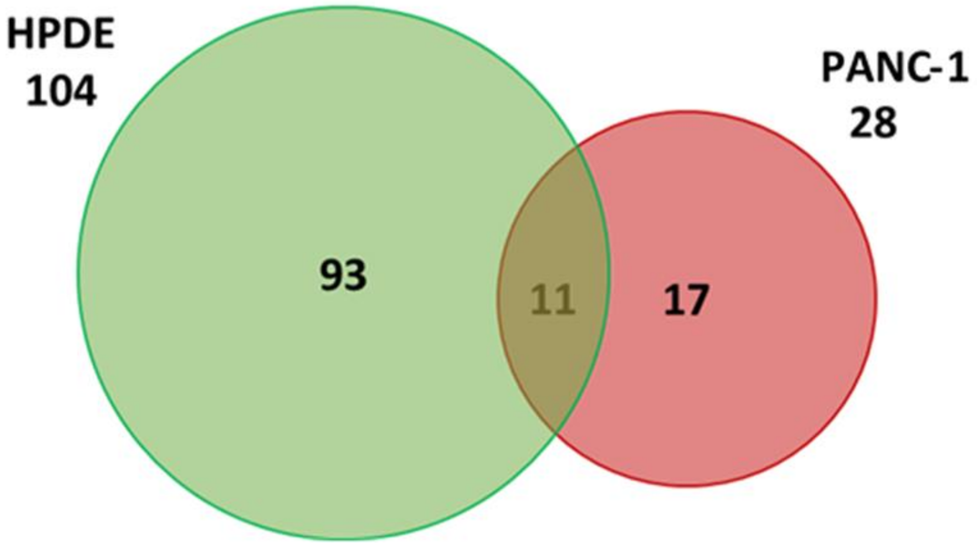

Figure S2

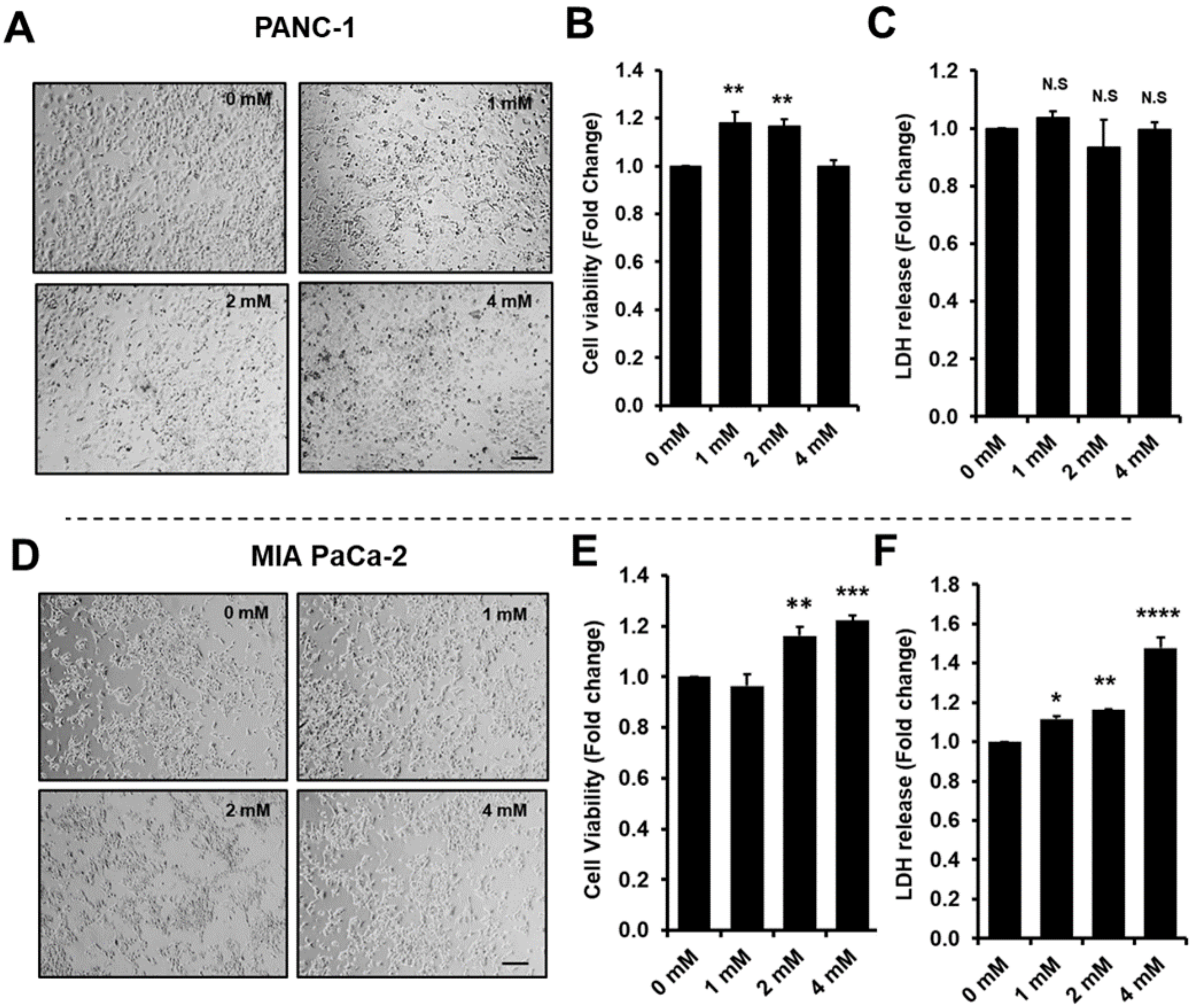

A
PANC-1
0 mM
1 mM
2 mM
4 mM
B
Cell viability (Fold Change)
1.4
1.2
1.0
0.8
0.6
0.4
0.2
0.0
**
**
0 mM
1 mM
2 mM
4 mM
C
LDH release (Fold change)
1.2
1.0
0.8
0.6
0.4
0.2
0.0
N.S
N.S
N.S
0 mM
1 mM
2 mM
4 mM
D
MIA PaCa-2
0 mM
1 mM
2 mM
4 mM
E
Cell Viability (Fold change)
1.4
1.2
1.0
0.8
0.6
0.4
0.2
0.0
**
***
0 mM
1 mM
2 mM
4 mM
F
LDH release (Fold change)
1.8
1.6
1.4
1.2
1.0
0.8
0.6
0.4
0.2
0.0
*
**
****
0 mM
1 mM
2 mM
4 mM

Figure S3

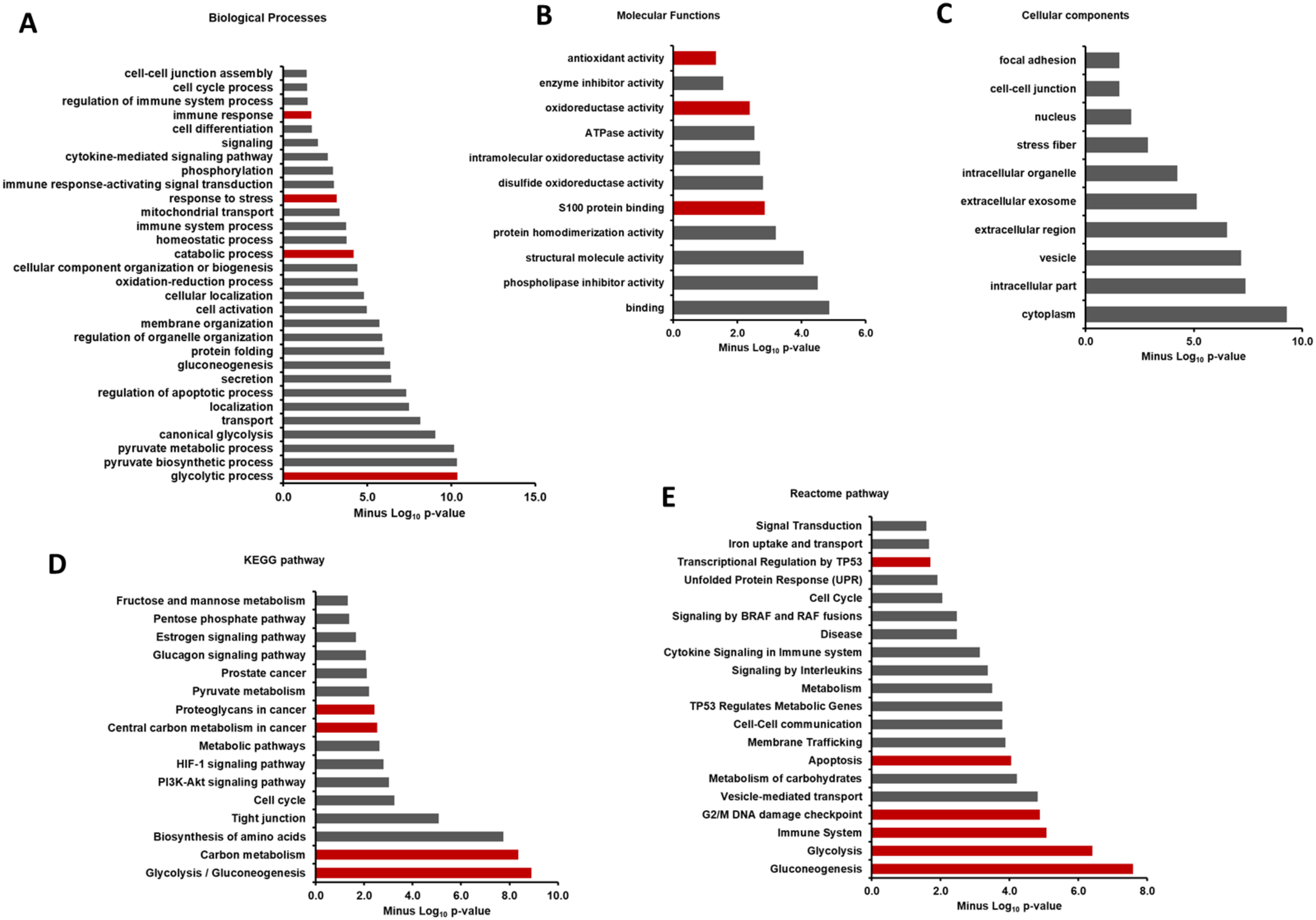
A
Biological Processes
cell-cell junction assembly
cell cycle process
regulation of immune system process
immune response
cell differentiation
signaling
cytokine-mediated signaling pathway
phosphorylation
immune response-activating signal transduction
response to stress
mitochondrial transport
immune system process
homeostatic process
catabolic process
cellular component organization or biogenesis
oxidation-reduction process
cellular localization
cell activation
membrane organization
regulation of organelle organization
protein folding
gluconeogenesis
secretion
regulation of apoptotic process
localization
transport
canonical glycolysis
pyruvate metabolic process
pyruvate biosynthetic process
glycolytic process
0.0
5.0
10.0
15.0
Minus Log10 p-value
B
Molecular Functions
antioxidant activity
enzyme inhibitor activity
oxidoreductase activity
ATPase activity
intramolecular oxidoreductase activity
disulfide oxidoreductase activity
S100 protein binding
protein homodimerization activity
structural molecule activity
phospholipase inhibitor activity
binding
0.0
2.0
4.0
6.0
Minus Log10 p-value
C
Cellular components
focal adhesion
cell-cell junction
nucleus
stress fiber
intracellular organelle
extracellular exosome
extracellular region
vesicle
intracellular part
cytoplasm
0.0
5.0
10.0
Minus Log10 p-value
D
KEGG pathway
Fructose and mannose metabolism
Pentose phosphate pathway
Estrogen signaling pathway
Glucagon signaling pathway
Prostate cancer
Pyruvate metabolism
Proteoglycans in cancer
Central carbon metabolism in cancer
Metabolic pathways
HIF-1 signaling pathway
PI3K-Akt signaling pathway
Cell cycle
Tight junction
Biosynthesis of amino acids
Carbon metabolism
Glycolysis / Gluconeogenesis
0.0
2.0
4.0
6.0
8.0
10.0
Minus Log10 p-value
E
Reactome pathway
Signal Transduction
Iron uptake and transport
Transcriptional Regulation by TP53
Unfolded Protein Response (UPR)
Cell Cycle
Signaling by BRAF and RAF fusions
Disease
Cytokine Signaling in Immune system
Signaling by Interleukins
Metabolism
TP53 Regulates Metabolic Genes
Cell-Cell communication
Membrane Trafficking
Apoptosis
Metabolism of carbohydrates
Vesicle-mediated transport
G2/M DNA damage checkpoint
Immune System
Glycolysis
Gluconeogenesis
0.0
2.0
4.0
6.0
8.0
Minus Log10 p-value

Figure S4

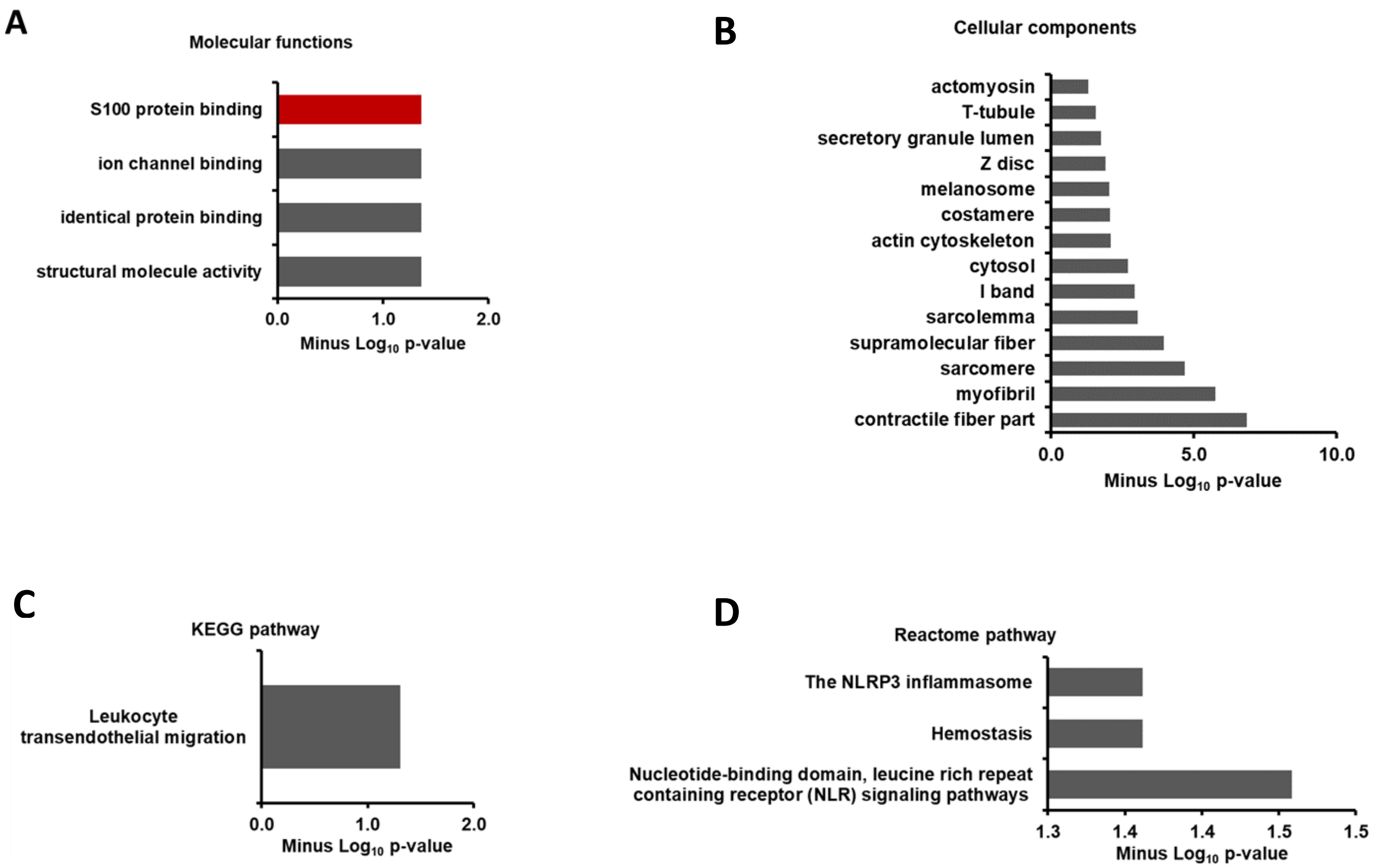
A
Molecular functions
S100 protein binding
ion channel binding
identical protein binding
structural molecule activity
0.0
1.0
2.0
Minus Log10 p-value
B
Cellular components
actomyosin
T-tubule
secretory granule lumen
Z disc
melanosome
costamere
actin cytoskeleton
cytosol
I band
sarcolemma
supramolecular fiber
sarcomere
myofibril
contractile fiber part
0.0
5.0
10.0
Minus Log10 p-value
C
KEGG pathway
Leukocyte transendothelial migration
0.0
1.0
2.0
Minus Log10 p-value
D
Reactome pathway
The NLRP3 inflammasome
Hemostasis
Nucleotide-binding domain, leucine rich repeat containing receptor (NLR) signaling pathways
1.3
1.4
1.4
1.5
1.5
Minus Log10 p-value
